# Skeletal muscle‐specific myostatin overexpression promotes muscle oxidative capacity and fatigue resistance in transgenic mice

**DOI:** 10.1113/EP093775

**Published:** 2026-05-28

**Authors:** Andy V. Khamoui, Andrea Abraham, Janos Porszasz, Istvan Kovanecz, Silvana Constantinescu, Harry B. Rossiter, Suzanne Reisz‐Porszasz

**Affiliations:** ^1^ Department of Exercise Science and Health Promotion Florida Atlantic University Boca Raton Florida USA; ^2^ Respiratory Research Center The Lundquist Institute for Biomedical Innovation at Harbor‐UCLA Medical Center Torrance California USA; ^3^ Department of Health and Life Sciences Charles R. Drew University of Medicine and Science Los Angeles California USA; ^4^ Division of Respiratory and Critical Care Physiology and Medicine Department of Medicine Harbor‐UCLA Medical Center Torrance California USA; ^5^ Department of Surgery Harbor‐UCLA Medical Center and The Lundquist Institute for Biomedical Innovation at Harbor‐UCLA Medical Center Torrance California USA; ^6^ Department of Urology David Geffen School of Medicine at UCLA Los Angeles California USA; ^7^ Science, Technology, Engineering and Mathematics Division Compton College Compton California USA

**Keywords:** metabolism, mitochondria, mouse, muscle fatigue, muscle physiology, oxidative phosphorylation

## Abstract

In addition to controlling muscle mass, myostatin may support oxidative metabolism and endurance. Loss of function through gene knockout or post‐natal blockade generally lowers muscle oxidative capacity and increases fatigability. These observations imply that myostatin activation could promote a more oxidative and less fatigable muscle phenotype. Gain‐of‐function approaches that activate myostatin in vivo, however, are largely absent. To test whether myostatin promotes oxidative metabolism in muscle, we constructed transgenic (TG) mice with myostatin (*Mstn*) gene overexpression restricted to skeletal muscle by inserting an *Mstn* cDNA construct under the MCK promoter to drive muscle‐specific expression of recombinant myostatin protein. On standard diet, TG had greater oxidative fibre expression, greater coupled maximal mitochondrial oxidative phosphorylation (OXPHOS), and increased in situ muscle fatigue resistance. Untargeted metabolomics identified greater stored carbohydrate and glycolytic intermediates in TG muscle, lower lactate/pyruvate and lower AMP that together indicate TG muscle to be energetically primed for carbohydrate‐fuelled OXPHOS. Further, TG had enhanced synthesis of spermidine, a polyamine and autophagy inducer implicated in mitochondrial quality control and geroprotection, and large changes in polyunsaturated fatty acid composition with reduced long‐chain saturated fat. When challenged by lipid overload, TG displayed some features of intolerance related to glucose clearance and contractility, but also compelling signs of resilience including maintenance of mitochondrial respiratory control and running critical power. Together, these data show that myostatin is not only a regulator of skeletal muscle mass but a central mediator of diverse metabolic pathways that reinforce muscle homeostasis and organismal resilience including carbohydrate metabolism, bioactive lipids, polyamine compounds and mitochondrial respiration.

## Abstract (highlights)

- **What is the central question of this study?**
  In myostatin loss‐of‐function experiments, muscle mass increases but oxidative metabolism diminishes: does myostatin confer a muscle oxidative phenotype?
- **What is the main finding and its importance?**
  Muscle‐specific myostatin overexpression promoted muscle fibre succinate dehydrogenase activity, maximal mitochondrial oxidative phosphorylation (OXPHOS) and muscle fatigue resistance, and metabolomics indicated energetic priming for carbohydrate‐fuelled OXPHOS, enrichment of spermidine involved in mitochondrial quality control, and reduced long‐chain saturated fats. The findings suggest that myostatin regulates not only muscle mass but also diverse metabolic pathways that reinforce muscle homeostasis.

## 1 INTRODUCTION

Myostatin was discovered in 1997 as a new member of the transforming growth factor‐β family that regulated skeletal muscle mass (McPherron et al., 1997). Known also as growth and differentiation factor‐8, myostatin is produced by skeletal muscle and secreted into blood where it circulates and acts locally on myofibres to restrict growth (Lee, 2023). Marked control of muscle mass by myostatin was demonstrated in knockout (KO) mice with constitutive myostatin (*Mstn*) gene deletion from embryonic development, and in animals carrying naturally occurring inactivating mutations of the *Mstn* gene (Grobet et al., 1997; McPherron & Lee, 1997; Mosher et al., 2007; Schuelke et al., 2004). All showed a hypermuscular phenotype due to myostatin deficiency. These observations subsequently sparked clinical interest in anti‐myostatin therapies for age‐related sarcopenia, muscular dystrophies and other diseases where muscle loss features prominently (Nielsen et al., 2021). There is ongoing interest in myostatin due to its evolving role in systemic metabolic regulation and cross‐talk with non‐muscle organs (Ongaro et al., 2025; Stefanakis et al., 2024; Wang et al., 2024).

Along with control of muscle mass, myostatin may be necessary to support muscle oxidative metabolism and endurance based on in vivo loss‐of‐function experiments. In *Mstn* KO mice, myostatin‐deficient muscles were bigger, sometimes stronger but more fatiguable and glycolytic and had less mitochondrial mass, lower mitochondrial oxidative phosphorylation (OXPHOS) and oxidative metabolism limitation (lower citrate synthase, β‐hydroxyacyl CoA dehydrogenase; and succinate‐positive fibres), which coincided with lower ${\dot{V}}_{O_{2}\max}$ and endurance (Amthor et al., 2007; Baati et al., 2017; Chabi et al., 2016; Giannesini et al., 2013; Girgenrath et al., 2005; Matsakas et al., 2015; Mendias et al., 2006; Mouisel et al., 2014; Ploquin et al., 2012; Wang et al., 2022). The increased muscle mass and fatigability and reduced oxidative metabolism in these null mice with *Mstn* deletion from very early life was recapitulated when myostatin deficiency was induced in adulthood (Mouisel et al., 2014; Welle et al., 2007).

When myostatin inhibition occurs during the lifecycle appears to be an important determinant of muscle phenotypes. Several lines of evidence indicate differences in the muscle structure and function responses that arise from genetic ablation of *Mstn* compared with post‐natal blockade in adulthood. While *Mstn* KO mice showed a slow‐to‐fast fibre type switch (Girgenrath et al., 2005), these changes were not seen in adult mice given a blocking antibody (Girgenrath et al., 2005), suggesting the presence of myostatin during prenatal development to direct fibre type specification. Proteome analysis gave further indication of such dependencies, where KO had >12‐fold more differentially regulated proteins in muscle and more hypertrophy compared to adult mice given myostatin blockade (Salzler et al., 2016).

Dependencies based on disease and type of stressor are also evident. Genetic or pharmacological myostatin antagonism in experimental model systems led to improved muscle phenotypes in ageing (Camporez et al., 2016), injury‐induced regeneration (McCroskery et al., 2005), Duchenne muscular dystrophy (Bogdanovich et al., 2002), cancer cachexia (Benny Klimek et al., 2010; Gallot et al., 2014), glucocorticoid treatment (Latres et al., 2015), amyotrophic lateral sclerosis (Morrison et al., 2009), nemaline myopathy (Lindqvist & Granzier, 2023), sex‐specific contractures (Emmert et al., 2022), spaceflight (Lee et al., 2020), and limb unloading and immobilization (Hanson et al., 2023; Latres et al., 2015). In contrast, myostatin inhibition showed limited efficacy in certain inherited neuromuscular diseases like spinal muscular atrophy (Sumner et al., 2009), limb‐girdle muscular dystrophy (Kramerova et al., 2020) and critical illness such as sepsis (Morel et al., 2024).

To further study *Mstn* function, myostatin‐deficient mice have been challenged by high‐fat (HF) diet to induce nutrient excess and metabolic stress. In HF mice with *Mstn* KO or transgenic expression of the inhibitory propeptide, there was increased lean mass, decreased fat mass, greater glucose clearance and resistance to obesity (Guo et al., 2009; Zhao et al., 2005). Likewise, adeno‐associated virus‐mediated myostatin inactivation or blocking peptibody in HF adult mice prevented fat mass gain and increased insulin sensitivity and muscle glucose clearance (Dong et al., 2016; Eilers et al., 2020), suggesting myostatin inhibition in adulthood to also protect against obesity and metabolic disease (Guo et al., 2012; McPherron & Lee, 2002).

The current understanding of myostatin biology has been largely derived from loss‐of‐function experiments. Gene KO or myostatin inhibition generally reduces muscle oxidative capacity and increases fatigability (Mouisel et al., 2014). This suggests that myostatin activation may promote a more oxidative and less fatigable muscle phenotype. Gain‐of‐function approaches that activate myostatin, however, are largely absent with some exceptions such as the implantation of Chinese hamster ovary cells in mice (Zimmers et al., 2002). This circulating overexpression model caused severe muscle and fat wasting stemming from supraphysiological levels of active myostatin at circulating concentrations unlikely to represent normal ranges (Lee, 2023; Zimmers et al., 2002).

Alternatively, we previously achieved transgenic *Mstn* overexpression restricted to skeletal muscle and heart, or only skeletal muscle, in vivo by inserting an *Mstn* cDNA construct in mice and driving muscle‐specific expression of recombinant myostatin protein with the MCK promoter (Artaza et al., 2007; Reisz‐Porszasz et al., 2003). *Mstn* TG mice express myostatin protein in skeletal muscle at ∼40–65% over wild‐type (WT) controls. In this model, *Mstn* TG mice are ∼10% lighter, have ∼20% smaller limb muscle mass and lower cardiac mass but unaltered systolic function (Artaza et al., 2007; Reisz‐Porszasz et al., 2003). Sex specificity was also observed, with these features occurring in male but not female TG mice (Reisz‐Porszasz et al., 2003).

In this investigation, *Mstn* TG mice were fed regular chow or HF diet to study the metabolic consequences of selective myostatin overexpression in skeletal muscle. Assessments included body composition, insulin sensitivity, running endurance, in situ mitochondrial respiration, isolated whole muscle fatigability, and gene and metabolome profiling. Based on myostatin inhibition generally reducing muscle oxidative capacity and increasing fatigability, we hypothesized that myostatin overexpression in skeletal muscle would promote muscle oxidative metabolism and endurance. Further, based on higher muscle oxidative capacity generally enabling the flexibility to adapt substrate use toward lipid oxidation during lipid oversupply (i.e., metabolic flexibility) (Dube et al., 2014; Goodpaster & Sparks, 2017; Muoio, 2014), we hypothesized that TG mice overexpressing myostatin would exhibit phenotypes consistent with tolerance to HF diet‐induced lipid overload.

## 2 METHODS

### 2.1 Ethical approval

All animals in this project were handled humanely in accordance with standards set forth by the Animal Welfare Act and other Federal, State and Local statutes and regulations relating to Charles R. Drew University of Medicine and Science IACUC approval (protocol No. I‐1 108–264).

### 2.2 Animals and design

WT C57BL/6 male and female mice were purchased from Harlan Sprague Dawley (Indianapolis, IN, USA; acquired by Inotiv, West Lafayette, IN, USA). KO BALB/c male and female mice were purchased from Taconic Bioscience, Inc. (Germantown, NY, USA). TG mice were generated in our laboratory and bred in‐house at the Charles R. Drew University vivarium. A detailed description of TG mouse construction was published previously (Reisz‐Porszasz et al., 2003). Briefly, to generate TG on C57BL/6 background, *Mstn* cDNA was cloned and sequenced from mouse skeletal muscle. The 1.1 kb sequence was subcloned into the pEGFP‐1 vector by substituting the EGFP sequence with *Mstn* sequence. The MCK (muscle‐specific creatine kinase) promoter was cloned into this construct. The MCK promoter‐Mstn cDNA‐polyA construct (2.6 kb) was released from the vector and used for mouse pronuclei injection after testing myostatin protein expression in vitro in C2C12 cells. Next, the purified transgene sequences were sent to the UC Irvine Transgenic Mouse Facility, and 300 pronuclei were injected with the DNA construct and transplanted into CB6F1 mice. Transgenic animals were identified by PCR of tail DNA. The size of the PCR product was 290 bp using the 5′ primer located at −209 bp upstream and the 3′ primer located at +60 bp downstream of the transcriptional start site. These primers overlap the joint sequences at the MCK promoter 3′ end and the *Mstn* gene 5′ end. Female and male animals carrying the transgene and their age‐matched controls were sacrificed at 7 weeks of age for genotyping and measurement of myostatin expression (*n* = 10 animals/group). Genotyping of mice was performed by Southern blot analysis of 30 µg *Eco*RI‐digested genomic DNA prepared from the liver, and Mstn expression was determined by RT‐PCR and western blotting (GDF‐8 antibody (1L4): sc‐134345 as primary Ab, and anti‐mouse IgG‐HRP: sc‐2055 as secondary Ab, Santa Cruz Biotechnology, Inc., Dallas, TX, USA).

Mice on regular chow (RG) and HF diets were used at 3–12 months depending on the experiment and assessed for body composition, insulin sensitivity, running endurance, mitochondrial respiration, muscle fatigability, and transcript and metabolite profile. All mice were housed individually in Charles R. Drew University's Vivarium individually at 22°C with a 12 h light period per day. All surgeries were performed using aseptic techniques in accordance with the NIH *Guide for the Care and Use of Laboratory Animals* and the Animal Welfare Act. Animals were anaesthetized with intraperitoneal injection of ketamine (30 mg/kg) and xylazine (5 mg/kg) and were monitored for heart rate, respiration and body temperature during surgery. In the fatigability experiments, 2.5% isoflurane anaesthesia was used. Adequate levels of anaesthesia were determined by toe pinch and the (lack of) reflex action. Animals were monitored daily for signs of surgical complications. Euthanasia was performed by carbon dioxide asphyxiation followed by cardiac puncture or isoflurane overdose (5%, fatigability experiment only) consistent with the Panel on Euthanasia of the American Veterinary Medical Association. After euthanasia, tissues were collected and placed either in formaldehyde solution or on dry ice and stored at −80°C for later processing.

### 2.3 Diets

Mice were allowed ad libitum access to water and chow. Standard chow (regular diet, RG) consisted of 10% kcal fat and metabolizable energy of 3.85 kcal/g (cat. no. D12450B, Research Diets Inc., New Brunswick, NJ, USA). HF diet contained 45% kcal fat and metabolizable energy of 4.73 kcal/g (cat. no. D12451, Research Diets) each for 4 weeks.

### 2.4 Voluntary ambulation

Ambulation was evaluated in WT, KO and TG mice by voluntary wheel running activity. The mouse running wheel (Columbus Instruments, Columbus, OH, USA) was placed into a normal home cage. The cage was equipped with a sensor that recorded the revolutions of the wheel. Mice were kept individually in the cage every second day (24 h duration) for 5 days on regular chow (*n* = 4–7/group, 6‐month males).

### 2.5 Echo MRI

In vivo body composition analysis was performed at the UCLA small animal imaging facility using Echo MRI (EchoMRI™ 3‐in‐1 Body Composition Analyzer, Houston, TX, USA), which allows measurements of fat mass and lean mass in live, awake animals (*n* = 5–8/group, 3‐month males). Experimental animals were transferred to UCLA in the early morning in a temperature‐controlled environment without restriction on food or drink. Mice were placed individually without anaesthesia or restraint into a closed plastic chamber that limits but does not restrict movement, and the NMR signal was applied, which generated contrast between fat and lean tissues. Scan time was ≤2–3 min/mouse.

### 2.6 Glucose and insulin tolerance tests

A glucose tolerance test (GTT) and insulin tolerance test (ITT) were administered to WT, KO and TG mice on regular and HF diets (*n* = 8/group, 3‐month males). Blood glucose was measured using a OneTouch ultra‐portable glucometer (Lifescan, Milpitas, CA, USA). For the insulin tolerance test, animals were fasted for 12 h. Insulin (0.75 U/kg) in 0.1 mL saline was injected i.p. into non‐anaesthetized mice. Blood samples were taken at 0, 10, 20 30, 45, 60 and 90 min for the determination of blood glucose. Animals were rescued with a 200 µL injection of 20% glucose and allowed immediate access to food once blood glucose levels reached 25 mg/dL. For the GTT, animals were fasted for 16 h. Morning‐fed glucose level was determined in duplicate from tail blood. Glucose was injected intraperitoneally (2 g/kg) into non‐anaesthetized mice. Blood samples were collected at 0, 10, 20, 30, 60, 90 and 120 min after glucose injection. Whole blood was collected following the glucose tolerance test by cardiac puncture after which animals were euthanized by CO_2_.

### 2.7 Maximal running speed and power–duration parameters

Whole animal exercise endurance was evaluated in WT, KO and TG mice on regular and HF diets using incremental and constant power tests (*n* = 8/group, 3‐month males). Following a short familiarization period, animals were placed on a motorized treadmill (TSE Systems, Berlin, Germany) to begin the incremental running test at 8 m/min for 5 min. Both speed and inclination angle were then increased, to 12 m/min and 5% increment, respectively, for 5 min. The next step was at 16 m/min, 10% increment for 5 min, with subsequent steps increasing by an additional 4 m/min and 5% increment. Animals were encouraged to comply with a 1.5 mA electric shock when contacting the back‐end of the treadmill belt, and the test was terminated when the animals could no longer avoid the electric shock grid despite manual encouragement from study personnel. Blood lactate was measured immediately before and after the incremental test and the maximal running speed recorded. Blood was taken from the tail vein, and one drop of blood was used for the lactate meter (Lactate Pro, ARKRAY, Inc., Kyoto, Japan). On separate testing days (at least 2 days apart), constant power tests were administered based on the maximal running speed obtained during the incremental test. Four speeds close to the top speed achieved during incremental testing (between 85% and 120%) were used to measure the exercise endurance (time to intolerance). Each running speed was tested on a different day. These tests were used to construct the hyperbolic relationship between power output and tolerable duration in the form: power output = critical power + (*W*′/tolerable duration). For this, power output was calculated for each mouse and combination of running speed and inclination, using: power output (mW) = body mass (kg) × 9.81 (m/s^2^) × speed (m/s) × sin(α) × 57.3, where α is the angle of the treadmill (α = arctan(%/100)). Critical power (CP) represents the highest running speed for which metabolic steady‐state can be achieved and the curvature constant (*W*′) describes the total work that can be performed above CP. Maximal running speed (m/min, from incremental testing), body mass normalized CP (mW/g) and body mass normalized *W*′ (mJ/g) are reported.

### 2.8 Fatigability

In situ muscle fatigability was assessed in the triceps surae of WT, KO and TG mice on regular and HF diets (*n* = 6–8/group, 3‐month males). Under isoflurane anaesthesia, each mouse was placed in the supine position on a temperature‐controlled metal platform. The left foot was fixed to an overhead metal rod and the knee immobilized. The triceps surae muscle complex was isolated, and the Achilles tendon was attached to a Grass FT03 force displacement transducer (Grass Technologies, Warwick, RI, USA) coupled with a Labtrax4‐24T data acquisition/stimulator device (World Precision Instruments, Sarasota, FL, USA) connected to a computer. The blood supply and innervations of the isolated muscles remained intact, and the physiological temperature/moisture maintained during the experiment by periodic rinse with 40°C saline solution. The sciatic nerve was stimulated with a bipolar platinum hook electrode using trains of 15 square wave 10 V pulses with 0.5 ms duration at 70 Hz. The stimulation duration was constant for 1 min followed by 1 min rest and was repeated three times for every incremental setting: 15, 30, 60 and 120 trains per minute. To normalize the muscle length changes, every measurement started from a 30 mN pre‐tension state. At the end of the experiment, mice were euthanized with isoflurane overdose.

### 2.9 High‐resolution respirometry

Mitochondrial respiration was measured in situ in WT, KO and TG mice on regular and HF diets (*n* = 6–8/group, 12‐month males). Freshly dissected medial gastrocnemius was mechanically separated in ice‐cold BIOPS into duplicate fibre bundles of ∼5 mg each, then permeabilized in BIOPS containing saponin (50 µg/mL) and incubated with gentle shaking on ice for 20 min. Following saponin treatment, fibre bundles were washed in respiration medium (MiR05) on ice with gentle shaking for 10 min. After washing, fibre bundles were blotted dry and weighed before placement into the respirometer chambers. Oxygen flux per tissue mass (pmol/s/mg) was recorded in real‐time at 37°C in the oxygen concentration range of 550–350 nmol/mL using high‐resolution respirometry (Oxygraph‐2k, Oroboros Instruments, Innsbruck, Austria). Respiration was assessed by the following sequential injections into the respirometer: (1) 1 mM malate, 75 µM palmitoyl‐carnitine, 10 mM glutamate and 2.5 mM ADP to achieve maximal phosphorylating respiration from electron input through complex I linked substrates (CI_P_) (with fatty acids via electron‐transferring flavoprotein); (2) 10 mM succinate to supply electrons into complex II to achieve maximal OXPHOS capacity from convergent electron flux through complex I and II (CI+II_P_); (3) 10 µM cytochrome *c* to assess outer mitochondrial membrane integrity, with samples not included when flux increased by >15% (Kuznetsov et al., 2008); (4) 0.5 µM carbonyl cyanide‐*p*‐trifluoromethoxyphenylhydrazone (FCCP) to assess complex I and II linked electron transport system (ETS) capacity (i.e., maximal capacity of the electron transfer system; CI+II_E_); (5) 0.5 µM rotenone to inhibit complex I (CII_E_); and (6) 2.5 µM antimycin A to inhibit complex III and obtain residual oxygen consumption. Oxygen flux for each respiratory state was corrected by subtracting the residual oxygen consumption. To determine the flux control ratio of maximal OXPHOS to ETS (*P*/*E*), which expresses respiratory control independent of mitochondrial content, CI+II_P_ was divided by maximal electron transfer system capacity (CI+II_E_) as the reference state (Pesta & Gnaiger, 2012). In addition, complex I activity was calculated as 1 minus maximal ETS flux with rotenone (CII_E_ from step 6) (Burtscher et al., 2015). Fluxes from each duplicate measurement were averaged for analysis.

### 2.10 Histomorphometry

Muscle tissues were collected from WT, KO and TG and immediately fixed in 10% formalin after excision and embedded in paraffin. Ten‐micrometre sections from medial muscles were stained with haematoxylin and eosin (H&E) for determination of muscle fibre cross‐sectional area (CSA) (*n* = 5/group, 12‐month males). Staining for succinate dehydrogenase (SDH) activity and anti‐myosin heavy chain (MyHC)‐IIA (MABT840 clone SC‐71, Sigma‐Aldrich, St Louis, MO, USA) was also performed to assess oxidative metabolic phenotype (*n* = 4/group, 12‐month males). Slides were analysed with an Olympus microscope using Stereologer software (MBF Bioscience, Williston, VT, USA). For CSA determination, sections were imaged at ×200 magnification, three microscopic fields were randomly selected and CSA measured from 200 fibres/field. CSA measurements for each genotype were binned according to following size distributions and presented as relative frequency: 0–499 µm^2^, 500–999 µm^2^, 1000–1499 µm^2^, 1500–1999 µm^2^, 2000–2499 µm^2^, 2500–2999 µm^2^ and >3000 µm^2^. For fibre oxidative metabolism, individual muscle fibres were classified based on SDH stain intensity as oxidative (dark stain), intermediate (mild stain) and non‐oxidative (unstained) (Blemker et al., 2024; Rivero et al., 1999). The percentage of each muscle fibre classifier (i.e., oxidative, intermediate, non‐oxidative) was determined via counts of 300–500 fibres in four to six regions from both the superficial and deep portions of the muscle.

### 2.11 Blood biochemistry

Blood (1 mL) was collected terminally from WT, KO and TG mice on regular and high fat diets (*n* = 5–7/group, 3‐month males) under deep anaesthesia by cardiac puncture from the right ventricle with a 25‐gauge needle and dispensed into microtubes. To obtain serum, samples were sat at room temperature for 30 min and allowed to clot before centrifugation at 1300 RCF for 15 min. Serum was collected, aliquoted and stored at −80°C until biochemical analysis. Samples were sent to the Mouse Metabolic Phenotyping Center at the University of Cincinnati (Cincinnati, OH, USA) and serum leptin, adiponectin, free fatty acids, triglycerides, cholesterol and phospholipids were assayed colourimetrically with in‐house protocols (test no. C1052, C1057 and C1083).

### 2.12 Gene expression array

Total RNA was extracted from the gastrocnemius muscle, and 1.5 µg RNA was used as input for cDNA synthesis. To explore differential regulation of gene expression based on myostatin expression, an array‐based platform was used to measure muscle transcripts in WT, KO and TG (*n* = 5/group, 3‐month males). The panel comprised 84 key genes representative of 18 different signal transduction pathways and was carried out in accordance with the manufacturer's protocol (Signal Transduction Pathway Finder RT^2^ profiler PCR arrays, cat. no. PAHS‐014A‐2, SABiosciences Corporation, Frederick MD, USA (acquired by Qiagen)). *Hsp90ab1* and *Gapdh* were used as normalization controls. Fold‐differences between TG/WT and KO/WT were done using the $2^{-\Delta C_{t}}$ method. Fold‐differences in TG/WT and KO/WT were considered upregulated when >2, and downregulated when <0.5.

### 2.13 Metabolomics

Untargeted metabolomics was performed by Metabolon (Durham, NC, USA) using GC/MS and LC/MS platforms to detect and quantify muscle metabolites in WT and TG mice on regular and HF diets (*n* = 5/group, 4–7‐month‐old males). TG mice were prioritized for this experiment and did not include KO. Sample extraction was done on frozen gastrocnemius muscle tissues using an automated workflow (MicroLab STAR® system, Hamilton Company, Reno, NV, USA). The extract was divided into two aliquots, one for GC and one for LC analysis. Samples allocated to LC/MS were analysed with Waters ACQUITY ultra‐performance liquid chromatography (Waters Corporation, Milford, MA, USA) and a Thermo‐Finnigan (San Jose, CA, USA) linear trap quadrupole (LTQ) mass spectrometer equipped with electrospray ionization source and linear ion‐trap mass analyser. The sample extract was split into two aliquots, dried, then reconstituted in acidic or basic LC‐compatible solvents, each containing 11 or more injection standards at fixed concentrations. One aliquot was analysed under acidic positive ion optimized conditions and the other under basic negative ion optimized conditions in two independent injections on separate dedicated columns. Extracts reconstituted in acidic conditions were gradient eluted using water and methanol both containing 0.1% formic acid, while the basic extracts contained 6.5 mM ammonium bicarbonate. The MS analysis alternated between MS and data‐dependent MS^2^ scans using dynamic exclusion. Samples analysed by GC/MS were re‐dried under vacuum desiccation for a minimum of 24 h prior to being derivatized under dried nitrogen using bistrimethyl‐silyl‐triflouroacetamide. The GC column was 5% phenyl, and the temperature ramp was from 40° to 300°C in a 16‐min period. Samples were analysed on a Thermo‐Finnigan Trace DSQ fast‐scanning single‐quadrupole mass spectrometer using electron impact ionization. Sample quality control was performed in parallel with in‐house methods by analysing pooled matrix samples, process blank, solvent blank and internal standards to assess batch effects, potential contamination, instrumentation performance and extraction quality. Instrument and process variability was 5% and 12%, respectively, which met in‐house acceptance criteria. Data extraction of raw mass spectrometry files, filtering based on quality control limits and metabolite identification was performed using Metabolon's in‐house workflow. Data were rescaled to set the median equal to 1 and missing values if any imputed with the minimum. Data dimensionality reduction was performed using two‐component principal component analysis (generated using Array Studio, OmicSoft). Two‐way ANOVA was used to identify metabolites exhibiting significant interaction and main effects at *P *< 0.05 for the experimental factors of genotype and diet. ANOVA contrasts were used to identify biochemicals that differed significantly among experimental groups at false discovery rate <0.05. Fold‐differences among groups were increased and decreased if the metabolite ratio was >1 and <1, respectively. Enrichment analysis on significantly changed metabolites was done with Metaboanalyst 6.0.

### 2.14 Statistical analysis

Normality was assessed prior to analysis by visualizing data distributions using histograms and *q–q* plots. To determine effects of myostatin expression status and diet on 28‐day body weight and food intake, tissue weights, running endurance, lactate response, in situ fatigability and mitochondrial respiration, two‐way ANOVA was used with genotype and diet as factors. For MRI‐based body composition, measurements made before and after diet were analysed by factorial ANOVA (genotype, diet) with repeated measures on time. Significant interaction and main effects from factorial ANOVAs were followed up with pairwise comparisons using Šidák's *post hoc* test. For experiments in which genotype was the single factor (i.e., blood biochemistry, activity counts, histology), one‐way ANOVA with Šidák's *post hoc* test was used to identify differences among WT, KO and TG. For GTT and ITT, pre‐ and post‐diet measurements were analysed for each group within each individual time point by Student's paired *t*‐test with Bonferroni correction for multiple comparisons. Level of significance was *P *≤ 0.05.

## 3 RESULTS

### 3.1 Transgenic mice with constitutive expression of myostatin in skeletal muscle

**FIGURE 1:**
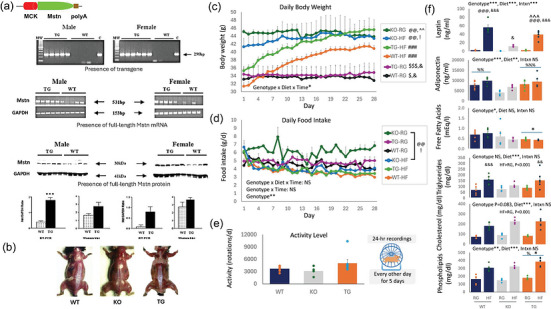
Transgenic mice with constitutive expression of myostatin in skeletal muscle. (a) MCK‐driven myostatin expression in skeletal muscle was determined by RT‐PCR and western blot. Agarose gel shows the presence of the transgene PCR products (290 bp), the full length of *Mstn* mRNA (531 bp), and western blot shows the expression on Mstn protein (30 kDa) from quadriceps muscles of WT and TG male and female mice. KO animals did not show any Mstn presence in their muscles. (b) Gross morphology of WT, KO and TG mice. (c) Body weight measured daily over 28 days in myostatin wild‐type (WT), knockout (KO) and transgenic (TG) mice on regular (RG) and high‐fat (HF) diets (*n* = 2–8/group, 6 months old). Data analysed with factorial ANOVA (genotype and diet with RM on time) using day 1 and day 28 as time points. Pairwise comparisons done with Šidák's *post hoc* test. *P < 0.05, interaction term. ^@@^ *P *< 0.01 vs. WT within same diet at day 1. ^^*P *< 0.01 vs. TG within same diet at day 1. ^!^ *P* = 0.052 vs. TG within same diet at day 1. ^###^ *P *< 0.001, day 28 > day 1. ^$^ *P *< 0.05, ^$$$^ *P *< 0.001, vs. HF diet within same genotype at day 28. ^&^ *P* = 0.063–0.116 vs. KO within same diet at day 28. (d) Food intake over 28 days. Data analysed with factorial ANOVA (genotype and diet with RM on time) using day 1 and day 28 as time points (*n* = 2–8/group). Pairwise comparisons done with Šidák's *post hoc* test. **P < 0.01, main effect of Genotype. ^@@^ *P *< 0.01, KO vs. WT. ^!^ *P* = 0.054, KO vs. TG. (e) Physical activity (wheel rotations) was monitored over 5 days. Data analysed as daily average during that period. 6‐month males, *n* = 4–7/group. Graphic from flaticon.com. (f) Blood biochemistry for lipid species and energy metabolism markers. Data analysed by 2‐way ANOVA with Šidák's *post hoc* test. Significant interaction term (Intxn) or main effects of Genotype/Diet are indicated at the top of each panel as *P < 0.05, **P < 0.01 and ***P < 0.001. NS, not significant. *n* = 6–8/group. ^@@@^ *P *< 0.001, different from KO within respective diet. ^&^ *P *< 0.05, ^&&^ *P *< 0.01, ^&&&^ *P *< 0.001, different from RG diet within genotype. ^^^^^ *P *< 0.001, different from WT within respective diet. *P < 0.05, main effect of Genotype: different from WT. ^%^ *P *< 0.05, ^%%^ *P *< 0.01, ^%%%^ *P *< 0.001, main effect of Genotype: different from KO.

TG mice had 37% increased *Mstn* mRNA in skeletal muscle by RT‐PCR, and 2.2‐fold elevated Mstn protein by immunoprobing with anti‐myostatin polyclonal antibody (Figure 1a). Gross morphological analysis showed hypermuscularity in KO, and slightly smaller but similar appearance in TG compared with WT (Figure 1b). Over 28 days, WT, KO and TG kept stable weight on the regular diet (Figure 1c), while KO was heavier than WT and TG on RG diet (Figure 1c). On the HF diet, WT and TG progressively gained weight whereas KO resisted weight gain (Figure 1c). Food consumed at the start and end of 28 days ranged from 4 to 7 g/day and was not significantly different between genotype and diet pairs (Genotype × Diet × Time, *P* = 0.669; and Genotype × Time, *P* = 0.237) (Figure 1d). Overall, KO consumed more food than WT and TG (Genotype main effect, *P* = 0.005) (Figure 1d). Spontaneous physical activity assessment over 5 days showed no difference between genotypes on running wheel activity counts (mean rotations per day) (Figure 1e), although a trend towards greater activity was observed in TG compared to WT (+36%, *P* = 0.392) and KO (+60%, *P* = 0.246) (Figure 1e). Blood biochemical analysis indicated no differences in leptin on RG diet (Figure 1f). On HF diet, all genotypes had greater leptin compared to RG, but this increase was attenuated in KO and TG compared to WT (Genotype × Diet, *P *< 0.001) (Figure 1f). In contrast, adiponectin concentrations in each genotype were independent of diet (Genotype × Diet, *P* = 0.706) (Figure 1f) but was greater overall in WT and TG compared to KO (Genotype effect, *P *< 0.001) (Figure 1f). Circulating lipids (i.e., free fatty acids, triglycerides, cholesterol, phospholipids) in each genotype were independent of diet (Genotype × Diet; free fatty acids: *P* = 0.564, triglycerides: *P* = 0.282, cholesterol: *P* = 0.435, phospholipids: *P* = 0.142). However, main effects of Genotype reflected lower free fatty acids in TG versus WT (*P* = 0.018) (Figure 1f), and greater phospholipids in TG compared to WT (*P* = 0.013) and KO (*P* = 0.022) (Figure 1f). As expected, large effects of diet were observed in which HF increased triglycerides, cholesterol and phospholipids (Diet main effect, all *P *< 0.001) (Figure 1f). Resistance to HF diet‐induced hypertriglyceridaemia was observed in KO, as WT and TG had greater triglycerides on HF compared to RG (WT: *P *< 0.001, TG: *P* = 0.004) (Figure 1f), whereas triglycerides in KO was not significantly difference between RG and HF (*P* = 0.196) (Figure 1f).

### 3.2 Oxidative fibre type predominance in skeletal muscle of TG mice

**FIGURE 2:**
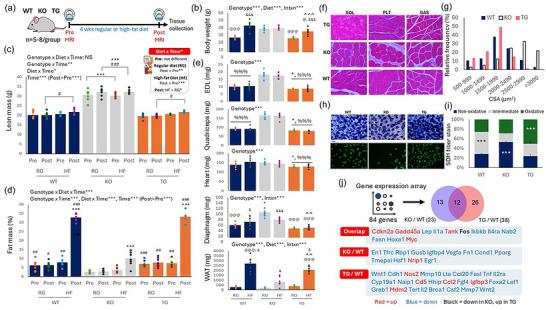
Oxidative fibre expression predominates in skeletal muscle of myostatin TG mice. (a) Body composition was determined in vivo by MRI in myostatin wild‐type (WT), knockout (KO) and transgenic (TG) mice on regular (RG) and high‐fat (HF) diets. (b) Body weight at sacrifice. ^@^ *P *< 0.05, ^@@^ *P *< 0.01, ^@@@^ *P *< 0.001, different from KO within respective diet. ^&^ *P *< 0.05, ^&&&^ *P *< 0.001, different from RG diet within genotype. ^^^^^ *P *< 0.001, different from WT within respective diet. (c) Absolute lean mass before (pre) and after (post) RG or HF diet. ***P < 0.001, greater than WT and TG within time point. ^#^ *P *< 0.05, ^###^ *P *< 0.001, greater than Pre within genotype. (d) Fat mass, expressed as a percentage of total body mass, before (pre) and after (post) RG or HF diet. ***P < 0.001, different from Pre within HF of each genotype. ^#^ *P *< 0.05, ^##^ *P *< 0.01, ^###^ *P *< 0.001, different from KO within diet and time point. (e) Tissue wet weight measurements taken at sacrifice. EDL, extensor digitorum longus; WAT, white adipose tissue. *P < 0.05, main effect of Genotype: less than WT. ^%%%^ *P *< 0.001, main effect of Genotype: less than KO. ^@^ *P *< 0.05, ^@@^ *P *< 0.01, ^@@@^ *P *< 0.001, different from KO within respective diet. ^&^ *P *< 0.05, ^&&&^ *P *< 0.001, different from RG diet within genotype. ^^^^ *P *< 0.01, different from WT within respective diet. (f) Transverse section of soleus (SOL), plantaris (PLT) and gastrocnemius (GAS) muscles stained with H&E for gross morphology. (g) Relative frequency of fibre cross‐sectional areas (CSA) derived from H&E staining of the gastrocnemius muscle in WT, KO and TG. (h) Transverse sections of the gastrocnemius stained with succinate dehydrogenase (SDH) as index of oxidative capacity (top row, blue signal, ×50), and anti‐myosin heavy chain IIA (bottom row, green signal, ×100). (i) Distribution of fibres based on SDH staining intensity as oxidative, intermediate and non‐oxidative in the gastrocnemius muscle. ***P < 0.001 vs. the other two genotypes for that fibre classification. (j) Expression array of 84 genes representing key signal transduction pathways showing differentially regulated genes that are shared and uniquely changed in KO and TG mice. All genes classified as upregulated had fold‐difference >2, and those downregulated <0.5. Data in panels (c, d) were analysed by 3‐way ANOVA and Šidák's *post hoc* test. Data in panels (b, e) were analysed by 2‐way ANOVA and Šidák's *post hoc* test. Significant interaction term (Intxn) or main effects are indicated at the top of each panel as *P < 0.05, **P < 0.01 and ***P < 0.001. NS, not significant. Mouse graphic from Free Clipart Library (clipart‐library.com) and MRI and diet graphics from flaticon.com.

**FIGURE A1:**
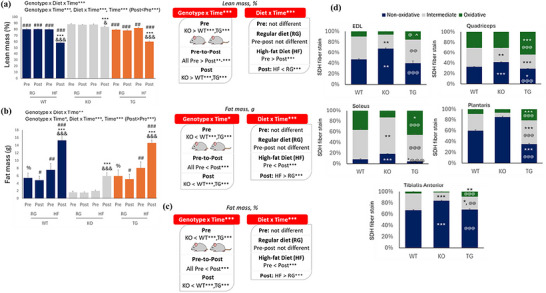
Body composition alterations by myostatin expression status and diet. (a) MRI determined lean mass expressed in relative terms as a percentage of total body mass. Symbols shown on left figure are follow‐up *post hoc* comparisons for the Genotype × Diet × Time interaction using Šidák's *post hoc* test. ***P < 0.001, different from Pre within HF of each genotype. ^##^ *P *< 0.01, ^###^ *P *< 0.001, different from KO within diet and time point. ^&^ *P *< 0.05, ^&&&^ *P *< 0.001, different from RG diet within genotype and time point. Inset panel on the right gives a summary of the Genotype × Time, and Diet × Time interactions for relative lean mass. (b) MRI determined absolute fat mass in grams. Symbols shown on left figure are follow‐up *post hoc* comparisons for the Genotype × Diet × Time interaction using Šidák's *post hoc* test. ***P < 0.001, different from Pre within HF of each genotype. ^#^ *P *< 0.05, ^##^ *P *< 0.01, ^###^ *P *< 0.001, different from KO within diet and time point. ^%^ *P* = 0.051–0.090, different from KO within diet and time point. ^&&&^ *P *< 0.001, different from RG diet within genotype and time point. Inset panel on the right gives a summary of the Genotype × Time, and Diet × Time interactions for absolute fat mass. (c) Summary of the Genotype × Time, and Diet × Time interactions for relative fat mass expressed as a percentage of total body mass. (d) Oxidative fibre distribution using succinate dehydrogenase (SDH)‐based classifications (oxidative/slow, intermediate/mixed and non‐oxidative/fast) in the EDL, quadriceps, soleus, plantaris and TA muscles of mice (12‐month males, *n* = 4/group). *P < 0.05, **P < 0.01, ***P < 0.001 vs. WT for that fibre type. ^@^ *P *< 0.05, ^@@^ *P *< 0.01, ^@@@^ *P *< 0.001 vs. KO for that fibre type. ^*P* = 0.109 vs. WT for that fibre type. Mouse graphic from Free Clipart Library (clipart‐library.com).

To assess consequences of myostatin expression status and diet on body composition, MRI was performed before and after diet in WT, KO and TG mice (Figure 2a). Body and tissue weights were then taken at sacrifice and morphology assessed on collected muscle tissues (Figure 2b,e). On the regular diet, KO mice were heavier than WT and TG (Figure 2b). On the HF diet, however, KO did not gain weight whereas WT and TG did (Figure 2b). Overall, TG mice were lighter than WT and KO (Genotype main effect, *P *< 0.001; TG < WT: *P* = 0.002 and TG < KO: *P *< 0.001) (Figure 2b). In the lean tissue compartment, lean mass change in each genotype was independent of diet (Genotype × Diet × Time, *P* = 0.546) (Figure 2c). At all time points, KO had more lean mass than WT and TG (Genotype × Time, *P *< 0.01) (Figure 2c), consistent with their well‐established hypermuscular phenotype. Over time, all genotypes showed an increase in lean mass (Genotype × Time, *P* = 0.002) (Figure 2c). Relative lean mass change (percentage of total mass), however, was diet dependent (Genotype × Diet × Time, *P *< 0.001) (Appendix Figure A1a). On the HF diet, all genotypes had significant loss of percentage lean mass over time (Appendix Figure A1a), but the degree of loss was less pronounced in KO (Appendix Figure A1a). In the fat compartment, fat mass change in both absolute and relative terms was diet dependent (Genotype × Diet × Time; absolute: *P* = 0.002, relative: *P *< 0.001) (Figure 2d and Appendix Figure A1b). While all genotypes did not change absolute or relative fat mass on RG diet (Figure 2d and Appendix Figure A1b), all genotypes significantly increased absolute and relative fat on HF diet, although KO gained significantly less than WT and TG (Figure 2d and Appendix Figure A1b,c).

Selected limb and cardiac muscle weights from each genotype were independent of diet (Genotype × Diet; EDL: *P* = 0.651, Quad: *P* = 0.891, Heart: *P* = 0.059, Diaphragm and WAT: *P *< 0.001) (Figure 2e). Weights of the EDL and quadriceps muscles were greater in KO compared to WT and TG (Genotype main effect, both *P *< 0.001) (Figure 2e), and lower in TG than WT (Figure 2e). The heart was smaller in TG compared to WT and TG (Figure 2e). Weights of the diaphragm and WAT were dependent on diet (Genotype × Diet, both *P *< 0.001) (Figure 2e). On a regular diet, KO had greater diaphragm weight than WT and TG (Figure 2e). On the HF diet, however, KO had lower diaphragm weight compared to regular diet (Figure 2e), whereas WT showed an increase and TG remained stable (Figure 2e). Overall, TG had a lighter diaphragm than WT and KO (Genotype main effect, *P *< 0.001; TG < WT and KO both *P *< 0.001) (Figure 2e). Visceral adiposity, as reflected by WAT weight, showed a dependency on diet (Genotype × Diet, *P *< 0.001) (Figure 2e). All genotypes increased WAT on the HF diet, but this occurred to a much lower extent in KO (Figure 2e).

Morphological analysis showed larger fibre CSAs in KO as expected (Figure 2f,g). WT and TG had similar fibre area profiles, although TG had a greater frequency of smaller fibres overall (Figure 2 g). For fibre oxidative metabolism, SDH staining and MHCIIA immunoreactivity on transverse sections showed signals consistent with lower muscle oxidative capacity in KO, and higher oxidative capacity in TG (dark blue and green, Figure 2h). Using SDH‐based fibre type inference, the gastrocnemius muscle of TG had the greatest proportion of oxidative fibres (Figure 2i), whereas KO expressed predominantly non‐oxidative fibres (Figure 2i). This expression pattern of oxidative fibre predominance in TG also extended to other hindlimb muscles (Appendix Figure A1d). For instance, mouse muscles considered typically fast fibre predominant (EDL, TA, plantaris), mixed (quadriceps, gastrocnemius) or slow (soleus) (Bloemberg & Quadrilatero, 2012) generally showed more SDH expression in TG (Appendix Figure A1d).

To explore KO and TG muscle at the transcript level, gene expression analysis was done with an array consisting of 84 key genes representing 18 different signal transduction pathways (Figure 2j). KO muscle had 25 changed transcripts and TG had 38, with 12 shared between them (Figure 2j). Of the 12 shared, the gene encoding the transcription factor c‐Fos was the only member regulated in opposite directions, with expression increased in TG muscle (Figure 2j). Other genes uniquely expressed in TG muscle with demonstrated impact on metabolism and/or inflammation include members of the Wnt pathway (*Wnt1*, *Wnt2*), *Igfbp3*, *Foxa2*, *Mdm2*, *Fgf4*, *Cd5* and *Ccl2*.

In summary, KO were heavier, leaner and had bigger muscles (Figure 2b,c,e–g). TG were lighter with some smaller muscles (EDL, quadriceps, heart, diaphragm) (Figure 2b,e). On HF diet, KO resisted weight gain (Figure 2b), relative lean tissue loss (Appendix Figure S1a), fat mass gain (Figure 2d,e and Appendix Figure S1b,c) and hypertriglyceridaemia (Figure 1f), consistent with published literature (Guo et al., 2009). On morphological analysis, SDH activity implied oxidative fibre type predominance and higher muscle oxidative capacity in TG (Figure 2 h,i and Appendix Figure A1d). At the molecular level, KO and TG mice had distinct transcript profiles (Figure 2j). The number of differentially expressed genes shared between genotypes accounted for <15% of the total gene expression panel (Figure 2j), and the transcription factor Fos the sole member regulated in opposite directions (Figure 2j).

### 3.3 Lower insulin sensitivity in myostatin overexpressing mice following lipid overload

**FIGURE 3:**
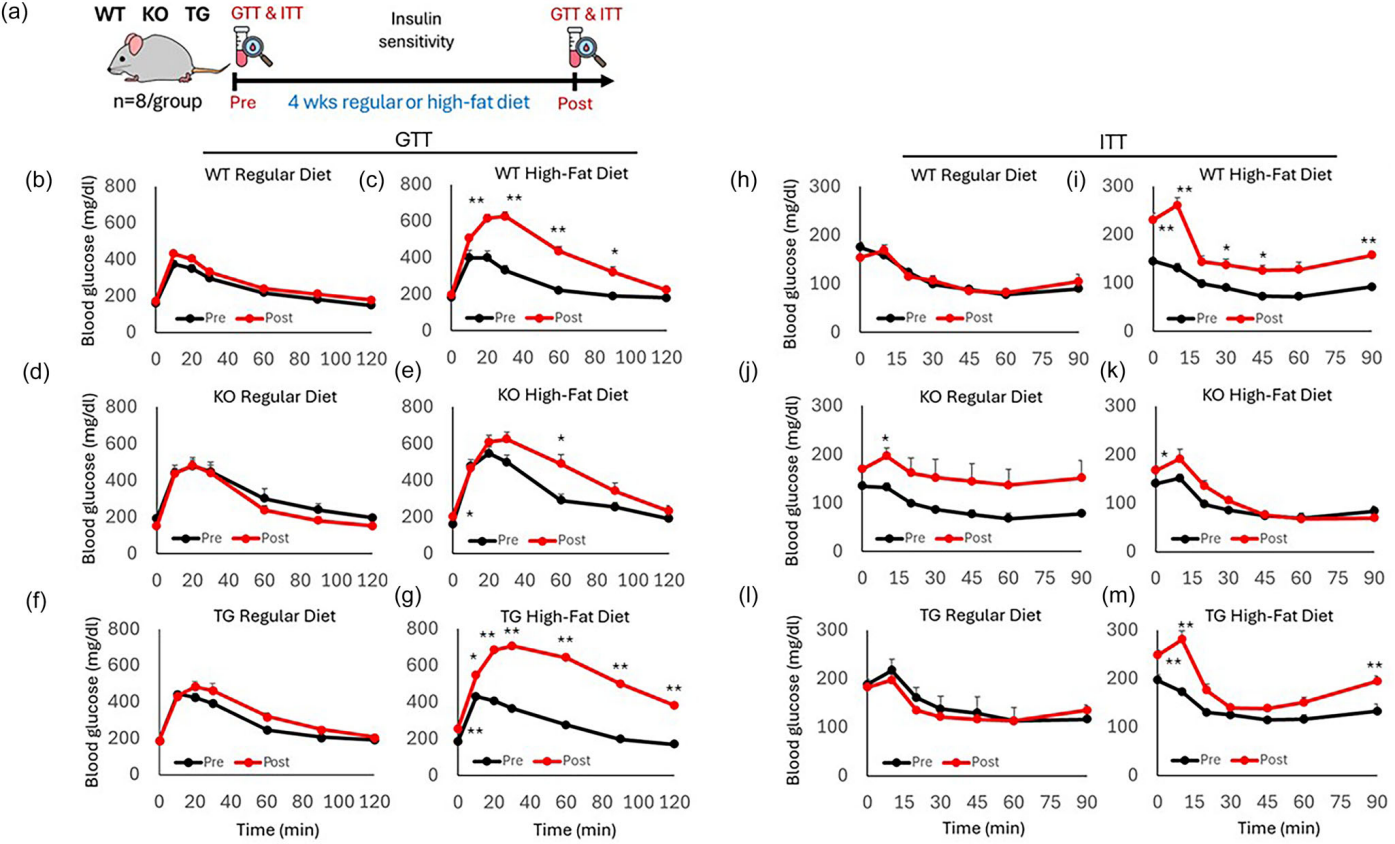
Lower insulin sensitivity in myostatin overexpressing mice following lipid overload. (a) To examine consequences of muscle myostatin expression status and diet on insulin sensitivity, glucose tolerance tests (GTT) and insulin tolerance tests (ITT) were administered before and after regular (RG) or high‐fat (HF) diet in myostatin wild‐type (WT), knockout (KO) and transgenic (TG) mice. (b, c) GTT in WT mice on RG or HF diets. (d, e) GTT in KO mice on RG or HF diets. (f, g) GTT in myostatin TG mice on RG or HF diets. (h, i) ITT in WT mice on RG or HF diets. (j, k) ITT in KO mice on RG or HF diets. (l, m) ITT in TG mice on RG or HF diets. For GTT and ITT, data analysed at individual time points within each genotype and diet combination by paired *t*‐tests and Bonferroni adjustment for multiple comparisons. *P ≤ 0.007, **P ≤ 0.001. Mouse graphic from Free Clipart Library (clipart‐library.com) and blood tube graphics from flaticon.com.

To examine effects of myostatin expression and HF diet on insulin sensitivity, glucose and insulin tolerance tests were administered before and after diet (Figure 3a). After regular diet, there were no differences in blood glucose clearance in any genotype (Figure 3b,d,f). After the HF diet, all genotypes had worse glucose tolerance, with blood glucose in KO elevated at 0 and 60 min (Figure 3e), in WT at 20, 30, 60 and 90 min (Figure 3c) and in TG at all time points up to 120 min (Figure 3 g). TG had the poorest glucose tolerance after short‐term exposure to HF diet (Figure 3 g). Insulin tolerance tests were largely in agreement (Figure 3h–m). After the regular diet, insulin response in all genotypes was mostly unchanged (Figure 3 h,j,l). After HF diet, WT showed a sharp decline in blood glucose at 20 min after insulin was given but remained elevated above pre‐diet level through 90 min (Figure 3i). KO response was mostly unchanged after HF diet (Figure 3k), consistent with resistance to the adverse effects of lipid overload. TG muscle showed signs of dampened insulin response at 10 and 90 min (Figure 3m) but was otherwise unchanged from pre‐diet responsiveness from 20 to 60 min (Figure 3m).

### 3.4 Myostatin overexpression supports endurance exercise tolerance during lipid overload

**FIGURE 4:**
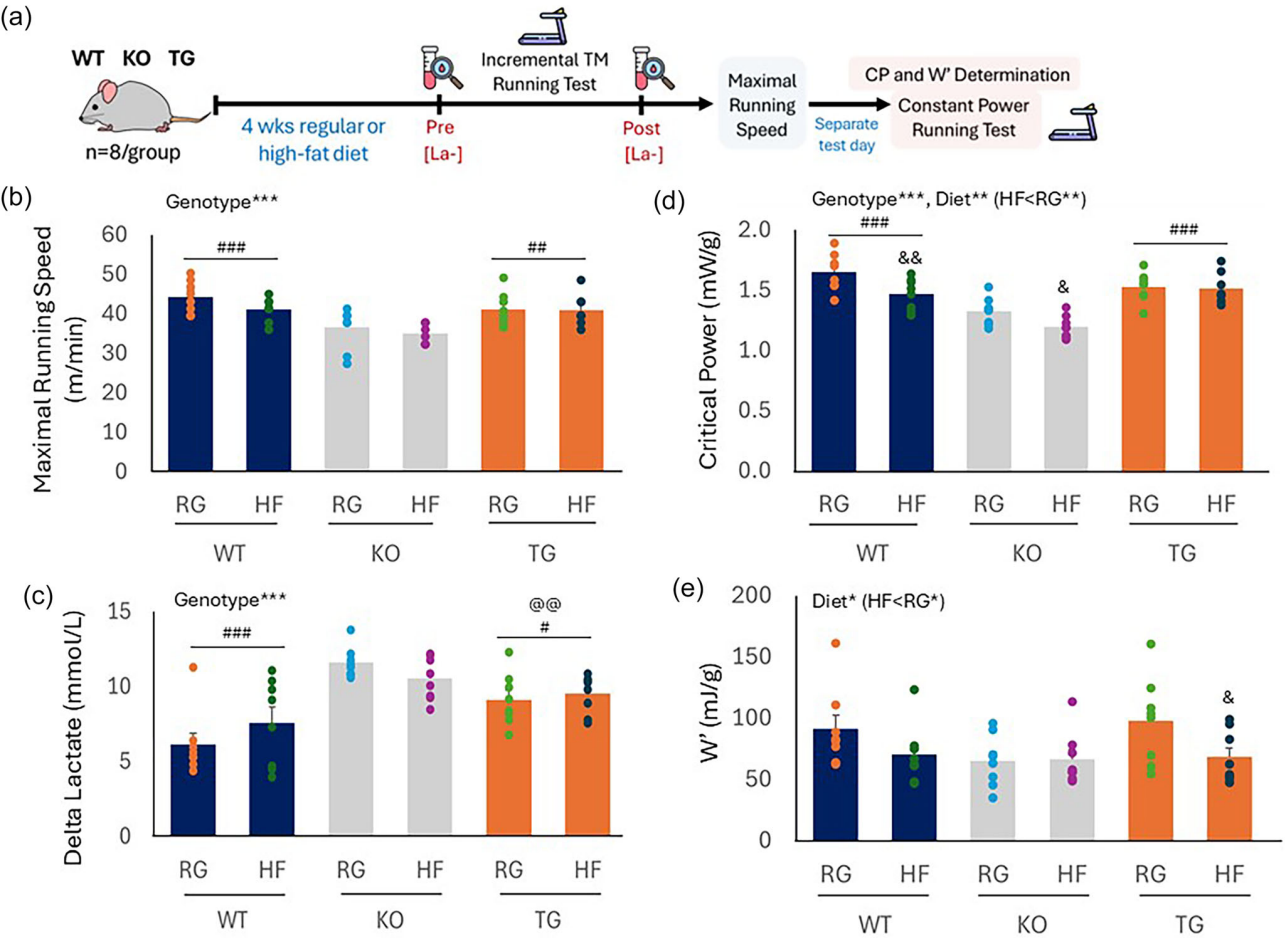
Myostatin overexpression supports endurance exercise tolerance following lipid overload. (a) Whole animal endurance was assessed in myostatin wild‐type (WT), knockout (KO) and transgenic (TG) mice on regular (RG) or high‐fat (HF) diet (3‐month males, *n* = 8/group). Blood lactate was measured before and after the incremental treadmill (TM) running test. Constant power tests were based on maximal running speed from the incremental test and used to derive body mass normalized critical power (CP) and work capacity above CP (*W*′). (b) Maximal running speed recorded during incremental running test. ***P < 0.001, main effect of Genotype. ^##^ *P *< 0.01, ^###^ *P *< 0.001 vs. KO, using Šidák's *post hoc* test to follow‐up the main effect of Genotype. (c) Blood lactate response to incremental running. Analysis performed on pre‐to‐post delta values. ***Main effect of Genotype significant at *P *< 0.001. Follow‐up pairwise comparisons with Šidák's *post hoc* test: KO greater than WT (^###^ *P *< 0.001) and TG (^#^ *P *< 0.05); TG greater than WT (^@@^ *P *< 0.01). (d, e) Critical power and *W*′ normalized to body mass. *P < 0.05, **P < 0.01, ***P < 0.001, main effect of Genotype or Diet. HF < RG at *P < 0.05, **P < 0.01, when following up main effects of Diet by Šidák's test. ^##^ *P *< 0.01, ^###^ *P *< 0.001 vs. KO, when following up the main effect of Genotype with Šidák's *post hoc* test. ^&^ *P *< 0.05, ^&&^ *P *< 0.01, different from RG diet within genotype by Šidák's test. Mouse graphic from Free Clipart Library (clipart‐library.com) and remaining graphics from flaticon.com.

To examine the influence of myostatin expression status and HF diet on whole animal endurance exercise, WT, KO and TG performed incremental and constant power running tests to obtain maximal speed, lactate response, CP and work capacity above CP (*W*′) (Figure 4a). Maximum running speed achieved in the incremental running test in each genotype did not depend on diet (Genotype × Diet, *P* = 0.561) (Figure 4b). Maximum running speed was lower overall in KO compared to WT and TG (Genotype main effect, *P *< 0.001) (Figure 4b). Blood lactate response did not depend on diet (Genotype × Diet, *P* = 0.164) but varied by genotype (Genotype main effect, *P *< 0.001) (Figure 4c). KO accumulated more lactate at peak exercise than WT and TG (Figure 4c), consistent with prior reports of glycolytic predominance in KO muscle (Mouisel et al., 2014). CP, which reflects the highest power output for which metabolism can achieve a steady state (Poole et al., 2016; Tiller et al., 2023), was lower in KO compared to WT and TG (Genotype main effect, *P *< 0.001) (Figure 4d). CP was also lowered overall by HF compared to RG (Diet main effect, *P* = 0.004) (Figure 4d), which in pairwise comparisons showed HF diet to lower CP in WT (*P* = 0.006) and KO (*P* = 0.041), but not TG (*P* = 0.825) (Figure 4d). Maintenance of CP suggests TG were better able to maintain endurance exercise tolerance during lipid overload. *W*′, which reflects work capacity above CP (i.e., available reserve), was reduced by HF diet (Diet main effect, *P* = 0.038) (Figure 4e). Pairwise comparisons showed HF to significantly lower *W*′ in TG (−30% vs. RG, *P* = 0.031), and a tendency to lower it in WT (−23% vs. RG, *P* = 0.123) but not KO (*P* = 0.919) (Figure 4e). In summary, KO had lower running speed, greater lactate accumulation during exercise and lower CP compared to WT and TG (Figure 4b,d), consistent with a more glycolytic phenotype. On HF diet, TG maintained their CP whereas WT and KO showed decrements (Figure 4d), consistent with a more oxidative phenotype during lipid overload.

### 3.5 Myostatin overexpression promotes OXPHOS capacity and fatigue resistance in muscle

**FIGURE 5:**
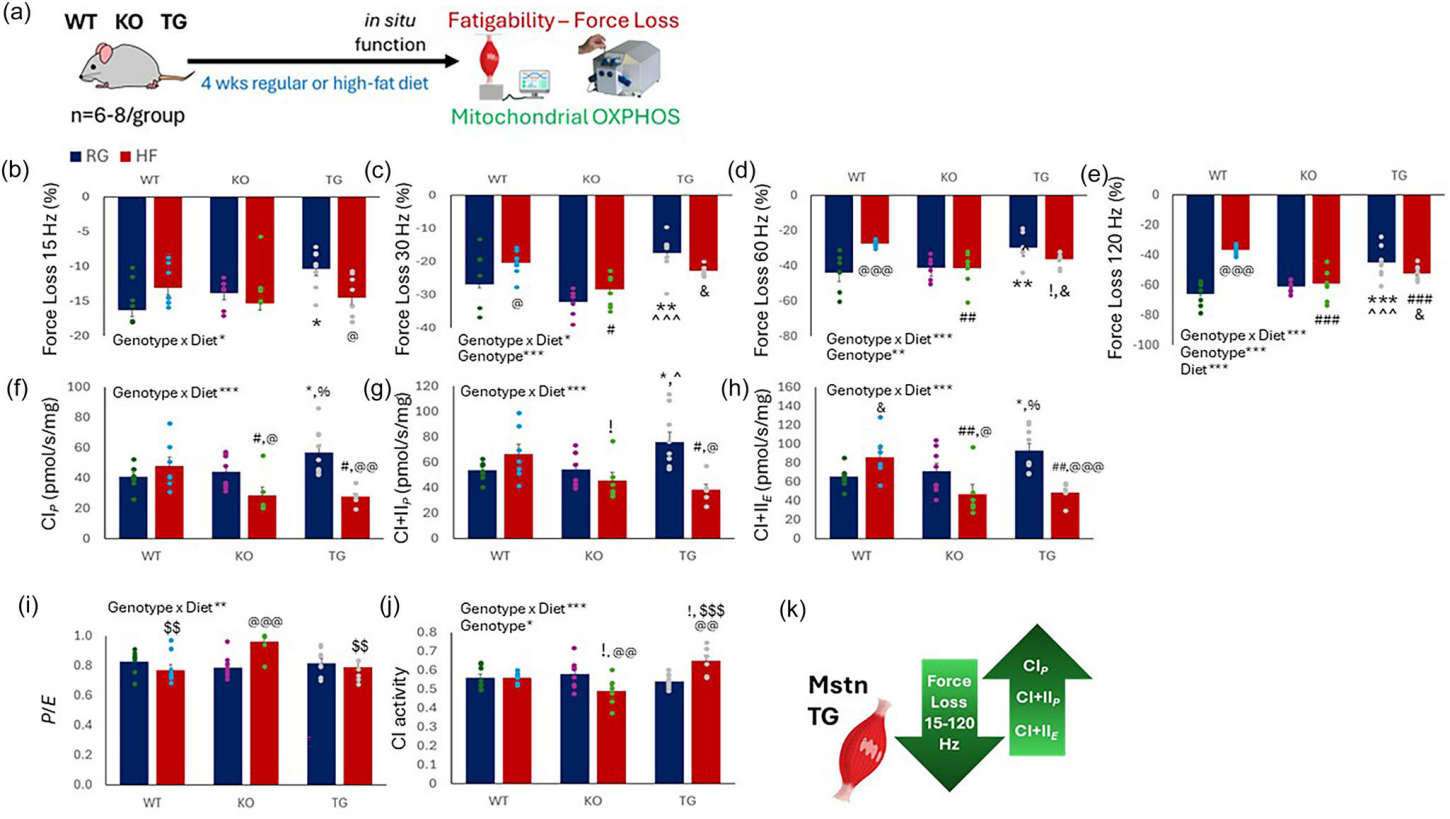
Myostatin overexpression promotes OXPHOS capacity and fatigue resistance in muscle. (a) In situ fatigability and mitochondrial respiration was assessed in myostatin wild‐type (WT), knockout (KO) and transgenic (TG) mice on regular (RG) or high‐fat (HF) diet. Fatigability was measured as force loss at different stimulation frequencies in the triceps surae complex (5‐month males, *n* = 6–8/group). Respiration was measured in permeabilized gastrocnemius fibre bundles by high‐resolution respirometry (12‐month males, *n* = 6–8/group). (b–e) Force loss at 15, 30, 60 and 120 Hz. (f–h) Mass‐specific oxygen flux (pmol/s/mg of muscle tissue) with 1 mM malate, 75 µM palmitoyl‐carnitine, 10 mM glutamate and 2.5 mM ADP to evaluate complex I linked OXPHOS (CI_P_) (f); addition of 10 mM succinate to evaluate complex I and II supported OXPHOS (CI+II_P_) (g); and addition of 0.5 µM FCCP to assess complex I and II linked electron transfer system (ETS) capacity (CI+II_E_) (h). (i) Flux control ratio of maximal OXPHOS to ETS (*P*/*E*), obtained by dividing CI+II_P_ by maximal electron transfer system capacity (CI+II_E_). (j) Complex I activity, calculated as 1 minus maximal ETS flux with 0.5 µM rotenone. (k) Working model in which resistance to fatigue in myostatin overexpressing muscle arises in part from enhanced mitochondrial respiration. Data analysed by 2‐way ANOVA and Šidák's *post hoc* test for paired comparisons. *P < 0.05, **P < 0.01, ***P < 0.001 vs. WT within RG diet. ^*P *< 0.05, ^^^*P *< 0.001 vs. KO within RG diet. ^%^ *P* = 0.09–0.10 vs. KO within RG diet. ^#^ *P *< 0.05, ^##^ *P *< 0.01, ^###^ *P *< 0.001 vs. WT within HF diet. ^!^ *P* = 0.052–0.084 vs. WT within HF diet. ^$$^ *P *< 0.01, ^$$$^ *P *< 0.001 vs. KO within HF diet. ^@^ *P *< 0.05, ^@@^ *P *< 0.01, ^@@@^ *P *< 0.001 vs. RG diet within genotype. ^&^ *P* = 0.052–0.073 vs. RG diet within genotype.

To assess consequences of myostatin expression and HF diet on in situ fatigability, force loss was measured in electrically stimulated triceps surae (Figure 5a). Percentage force loss in each genotype depended on diet (Genotype × Diet; 15 Hz: *P* = 0.036, 30 Hz: *P* = 0.015, 60 and 120 Hz: *P *< 0.001) (Figure 5b–e). On a regular diet, force loss was significantly lower in TG compared to WT (Figure 5b–e) and KO (Figure 5c,e), indicating less fatigability in TG muscle. On the HF diet, however, force loss was significantly greater in KO (Figure 5c–e) and TG (Figure 5e) compared to WT, suggesting susceptibility of KO and TG muscle to the adverse effects of lipid overload. For KO on HF diet, force loss was not different from that with the regular diet (Figure 5b–e), whereas TG on HF diet had significantly greater (Figure 5b) or a tendency for greater force loss (Figure 5c–e) than with the regular diet, implying greater deficit of TG muscle contractility following lipid overload.

To assess consequences of myostatin expression and HF diet on in situ muscle mitochondrial function, respiration was determined in permeabilized gastrocnemius fibre bundles. Mass‐specific respiration in each genotype depended on diet (Genotype × Diet, all *P *< 0.001) (Figure 5f–h). On a regular diet, TG muscle had greater complex I OXPHOS (Figure 5f), complex I+II linked OXPHOS (Figure 5g) and complex I+II linked ETS capacity (Figure 5h) compared to WT and KO. On the HF diet, however, these indicators of mass‐specific respiration were lower in KO and TG compared to WT (Figure 5f–h), suggesting susceptibility of KO and TG muscle respiration to lipid overload. For KO and TG but not WT, respiration on the HF diet was lower than on the regular diet (Figure 5f–h), indicating sensitivity of OXPHOS and ETS capacities in KO and TG muscle to lipid overload. When the flux control ratio of OXPHOS to ETS (*P*/*E*) was analysed (Figure 5i), the genotypes exhibited a dependency on diet (Genotype × Diet, *P *< 0.001), where KO on HF diet increased *P*/*E* while WT and TG did not (Figure 5i), suggesting better maintenance of coupling control in TG but not KO during lipid overload. When complex I activity was analysed from 1 – maximum ETS flux with rotenone (Figure 5j), WT had no change on the HF diet compared to regular diet (Figure 5j), whereas KO decreased and TG increased complex I activity (Figure 5j), consistent with more complex I activity in myostatin overexpressing TG muscle during lipid overload.

In summary, on a regular diet TG muscle had less force loss and greater mass‐specific OXPHOS than WT and KO, suggesting resistance to fatigue in TG muscle and enhanced mitochondrial respiration with myostatin overexpression (Figure 5k). On the HF diet, there was more force loss and lower mass‐specific OXPHOS in KO and TG (Figure 5b–h), suggesting muscle fatigability and respiratory limitation during lipid overload in myostatin deficient and overexpressing muscles. TG muscle contractility might be most sensitive to lipid overload, as TG tended to have more force loss in HF versus regular diet at all stimulation frequencies – a pattern that did not occur in KO or WT on HF diet (Figure 5b–e). Although TG muscle contractility may be susceptible to lipid overload, selected indicators of mitochondrial quality reflected better mitochondrial respiratory control in TG muscle when challenged by HF diet (Figure 5i–j).

### 3.6 Myostatin overexpression enhances carbohydrate metabolism in muscle

**FIGURE 6:**
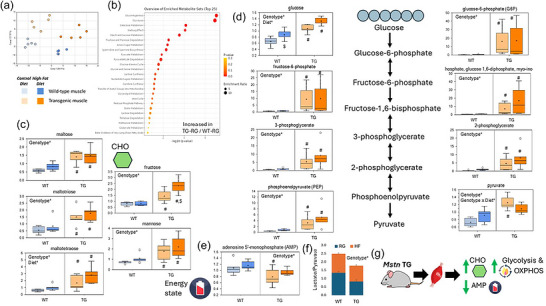
Carbohydrate‐fuelled oxidative metabolism in myostatin overexpressing muscle. Untargeted metabolomics was performed to obtain global muscle metabolites in myostatin wild‐type (WT) and transgenic (TG) overexpressing muscle under regular (RG) and high‐fat (HF) diets (4–7‐month males, *n* = 5/group). (a) Two‐component principal component analysis showed clear separation of muscle profiles by myostatin expression status and diet, reflecting the distinct molecular metabolism of transgenic, muscle‐specific myostatin overexpression. (b) Metabolites significantly increased in TG muscle on regular diet relative to WT (i.e., TG–RG/WT–RG) were input for enrichment analysis in Metaboanalyst, yielding glycolysis and carbohydrate metabolism as prominently enriched metabolite sets. (c) Individual metabolites in TG–RG/WT–RG showed several saccharides and glycogen breakdown products at greater levels in TG muscle. (d) Glycolytic pathway metabolites were detected at elevated levels in TG muscle, pointing to enhanced glycolytic activity. (e) AMP was lower in TG muscle consistent with less energetic stress. (f) Lactate to pyruvate ratio was also lower in TG muscle consistent with an oxidative‐like phenotype in myostatin overexpressing muscle. (g) Working model in which TG muscle on standard diet had greater intramuscular carbohydrate concentration and glycolytic flux, along with a lower lactate/pyruvate ratio, higher OXPHOS (from Figure 5) and lower AMP that together point to TG muscle being primed for oxidative metabolism of carbohydrates to sustain energy provision. Data analysed by 2‐way ANOVA and ANOVA contrasts to identify muscle metabolites that differed significantly between experimental groups at adjusted *P* < 0.05. *P ≤ 0.05, main effect of Genotype or Diet, or Genotype × Diet interaction. ^#^ *P *≤ 0.05, vs. WT within respective diet. ^$^ *P *≤ 0.05, vs. regular diet within respective genotype. + symbol within box plots represents mean value.

**FIGURE 7:**
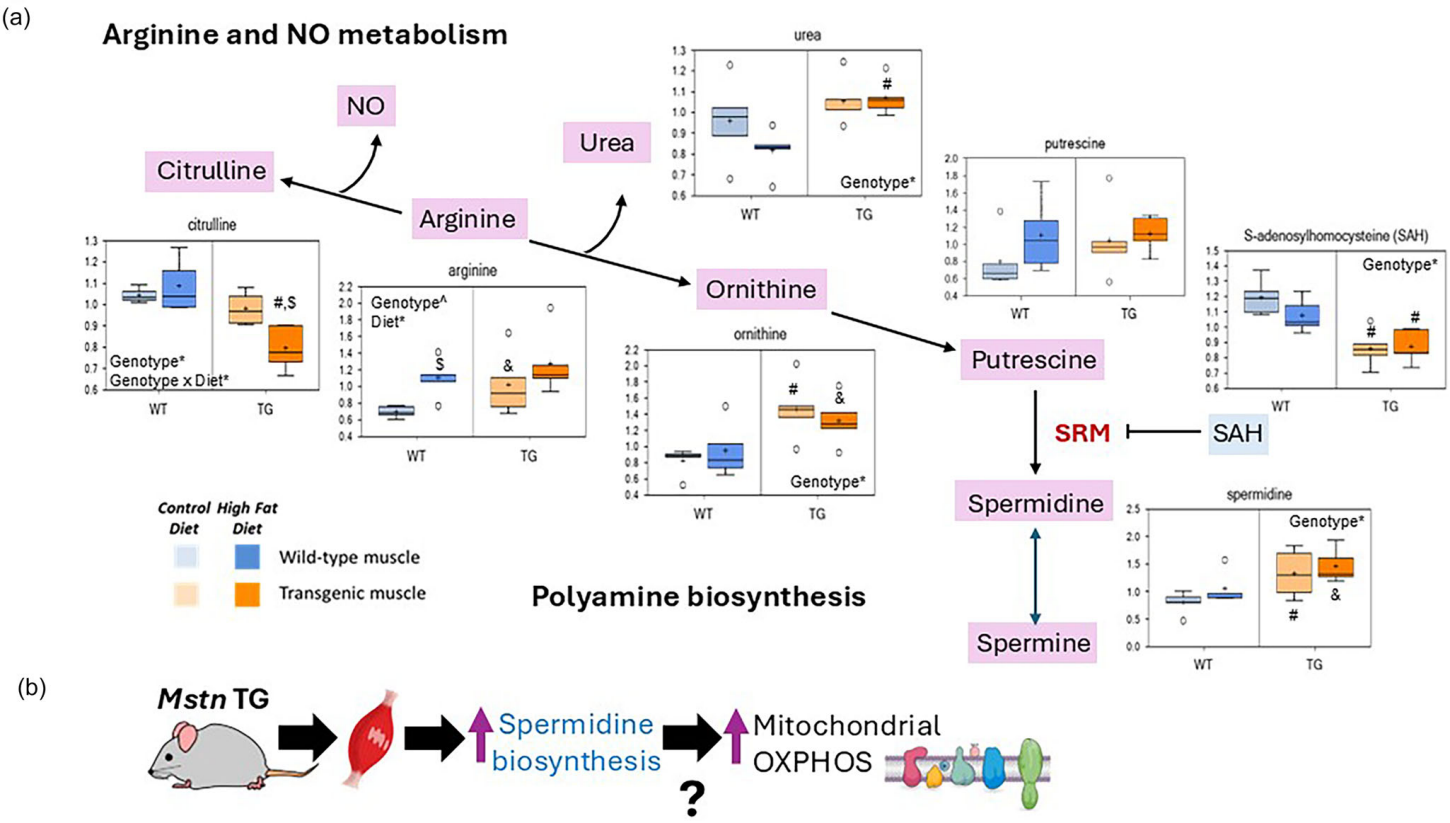
Enhanced spermidine biosynthesis in myostatin overexpressing muscle. Synthesis of spermidine and spermine, polyamines implicated in muscle and mitochondrial function, was an enriched metabolite set in TG muscle on regular diet (see Figure 6b), prompting further examination of individual metabolites comprising this pathway. (a) Arginine tended to increase but citrulline was decreased in TG muscle, suggesting that NO and citrulline production were not favoured in TG muscle. Instead, ornithine and the byproduct urea were both elevated along with increased spermidine, possibly from lower levels of *S*‐adenosylhomocysteine (SAH), an inhibitor of the spermidine synthase (SRM) enzyme that catalyses the formation of spermidine. (b) Working mechanism in which myostatin overexpressing muscle has enhanced spermidine biosynthesis in support of higher mitochondrial respiratory function. Metabolite data in panel (a) are from myostatin wild‐type (WT) and transgenic (TG) overexpressing muscle under regular (RG) and high‐fat (HF) diets (4–7‐month males, *n* = 5/group). Data analysed by 2‐way ANOVA and ANOVA contrasts to identify muscle metabolites that differed significantly between experimental groups at adjusted *P *< 0.05. ^#^ *P *≤ 0.05, vs. WT within respective diet. ^$^ *P *≤ 0.05, vs. regular diet within respective genotype. ^&^0.05 < *P *< 0.10, vs. WT within respective diet. *P ≤ 0.05, main effect of Genotype or Diet, or Genotype × Diet interaction. ^0.05 < *P *< 0.10, main effect of Genotype. + symbol within box plots represent mean values.

**FIGURE 8:**
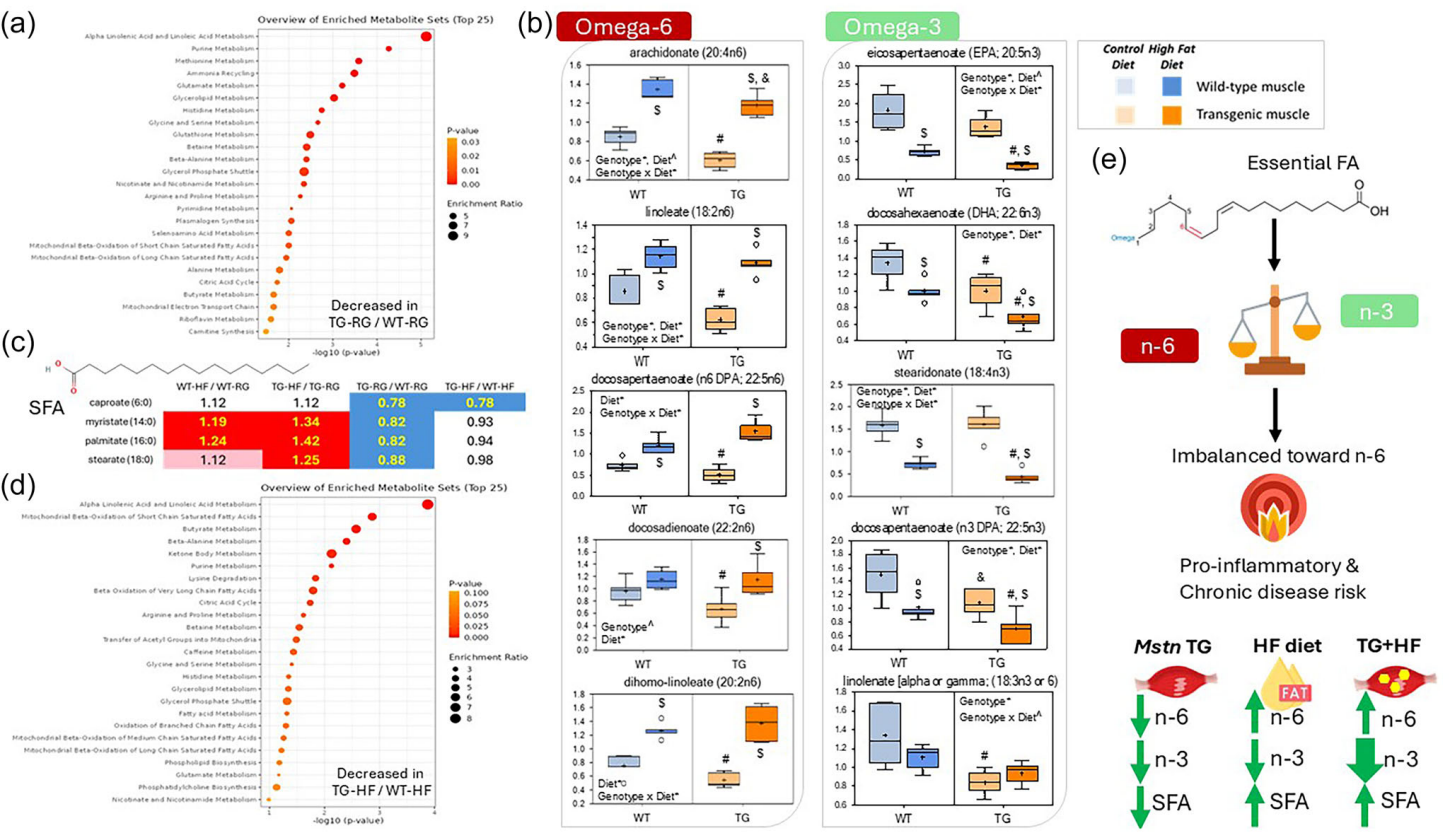
Myostatin overexpression modified fatty acid composition in muscle. Fatty acid profile of myostatin wild‐type (WT) and transgenic (TG) overexpressing muscle under regular (RG) and high‐fat (HF) diets (4–7‐month males, *n* = 5/group). (a) ‘Alpha linolenic acid and linoleic acid metabolism’ was the top enriched set when metabolites decreased in TG–RG/WT–RG were input for enrichment. (b) Omega‐6 and omega‐3 polyunsaturated fatty acids, bioactive lipid species well‐known to regulate multiple cellular processes, showed dependency based on myostatin expression status and/or diet. Omega‐6 and ‐3 fatty acids generally decreased in TG muscle (Genotype main effects, *P *< 0.05). (c) Saturated fatty acid (SFA) profile of TG muscle on RG and HF diets. Values are fold‐change for indicated comparison. Blue shade indicates significantly decreased, red shade means significantly increased and light red shade a tendency to increase. Several long‐chain saturated fatty acids consistently decreased in TG muscle on regular diet. (d) Enrichment on metabolites that decreased in TG muscle on high‐fat diet (TG–HF/WT–HF) also returned ‘alpha linolenic acid and linoleic acid metabolism’ as the top set. Here, the omega‐3 fatty acids decreased most (see panel b). (e) Summary of fatty acid alterations. The ratio of omega‐6/‐3 may reflect inflammatory status and chronic disease risk, where increased omega‐6/‐3 is pro‐inflammatory and unfavourable. Oversized arrow reflects omega‐3 fatty acids were depleted more severely in TG muscle when challenged by lipid overload, and this feature may distinguish TG muscle. Graphics in (e) from flaticon.com. Data analysed by 2‐way ANOVA and ANOVA contrasts to identify muscle metabolites that differed significantly between experimental groups at adjusted *P *< 0.05. #*P *≤ 0.05, vs. WT within respective diet. $*P *≤ 0.05, vs. regular diet within respective genotype. ^&^0.05 < *P *< 0.10, vs. WT within respective diet. ^%^0.05 < *P *< 0.10, vs. regular diet within respective genotype. *P ≤ 0.05, main effect of Genotype or Diet, or Genotype × Diet interaction. ^0.05 < *P *< 0.10, main effect of Genotype or Diet, or Genotype × Diet interaction. + symbol within box plots represents mean value.

**FIGURE A2:**
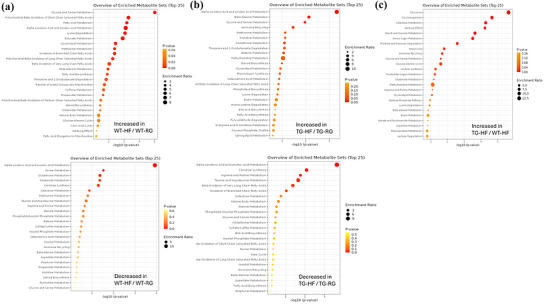
Enrichment analysis on differentially abundant muscle metabolites in myostatin TG mice. Enrichment analysis was performed by Metaboanalyst on differentially abundant metabolites in myostatin wild‐type (WT) and transgenic (TG) overexpressing muscle under regular (RG) and high‐fat (HF) diets (4–7‐month males, *n* = 5/group). (a) Enriched sets from muscle metabolites increased and decreased in WT–HF/WT–RG. (b) Enriched sets from muscle metabolites increased and decreased in TG–HF/TG–RG. (c) Enriched sets from muscle metabolites increased in TG–HF/WT–HF.

To obtain an unbiased molecular readout of metabolism, untargeted metabolomics was performed to derive global metabolite profiles in myostatin overexpressing muscle under regular and HF diet (Figures 6, 7, 8 and Appendix Figure A2). In the overall dataset, metabolomic analysis identified 270 compounds of known identity in muscle (Supporting information Table S1). For each of the four paired comparisons (i.e., WT–HF/WT–RG, TG–HF/TG–RG, TG–RG/WT–RG and TG–HF/WT–HF), the number of significantly increased metabolites ranged from 29 to 59, and the number of metabolites significantly decreased from 40 to 59 (Supporting information Table S1). Significantly changed metabolites due to Genotype main effect, Diet main effect and interaction term were 112, 118 and 32, respectively (Supporting information Table S1). Two‐component principal component analysis using global biochemical data showed a clear segregation of muscle profiles by myostatin expression status and diet (Figure 6a), highlighting the distinct molecular metabolism in this transgenic model of muscle‐specific myostatin overexpression. The diet effect was predominantly in component 1, which accounted for >25% of the variation in muscle, and the myostatin expression effect was in component 2, which accounted for >17% of the variation in muscle (Figure 6a).

To first examine the alterations occurring from transgenic overexpression of myostatin under normal diet, significantly changed metabolites (i.e., TG–RG/ WT–RG) were sorted and analysed individually or used collectively as input for enrichment analysis (Figure 6b–g). Increased metabolite sets pertained predominantly to glycolysis and carbohydrate metabolism (Figure 6b). Inspection of individual metabolites showed several saccharides and glycogen breakdown products at greater levels in TG muscle (Genotype main effects, *P *< 0.05) (Figure 6c). Similarly, glycolytic pathway intermediates were detected at elevated amounts in TG muscle (Genotype main effects, *P *< 0.05) (Figure 6d), accounting for the largest fold‐differences seen in the overall dataset and pointing to enhanced glycolytic flux. Adenosine 5′‐monophosphate (AMP) was lower in TG muscle (Figure 6e), suggesting less energetic stress, while the ratio of lactate to pyruvate was also lower (Figure 6f), implying a more oxidative phenotype in TG muscle. Overall, these data suggest that on a regular diet, TG muscle had greater carbohydrate concentration and ongoing glycolytic activity, lower lactate/pyruvate, greater OXPHOS (from Figure 5) and lower AMP (higher ATP), which together imply TG muscle to be primed for oxidative metabolism of carbohydrates to support muscle ATP supply (Figure 6g).

Another pathway enriched from the increased metabolites in TG muscle on regular diet was the ‘spermidine and spermine biosynthesis’ pathway (Figure 6b). This prompted further examination of metabolites comprising this pathway as spermidine and spermine are polyamines implicated in muscle phenotypes, autophagy, mitochondrial biogenesis, respiratory function and quality control (Al‐Habsi et al., 2022; Hofer et al., 2022; Madeo et al., 2018; Song et al., 2025; Tabbaa et al., 2021; Wang et al., 2020). Along with spermidine biosynthesis, this pathway is also linked to NO formation and citrulline through arginine (Madeo et al., 2018). In TG muscle, arginine increased but citrulline decreased (Figure 7a), suggesting that NO and citrulline production were not favoured in TG muscle. Instead, arginine appeared to be used for synthesis of ornithine and the byproduct urea, which were both elevated (Figure 7a). This ultimately enhanced spermidine biosynthesis (Figure 7a), which may be associated with lower levels of *S*‐adenosylhomocysteine (SAH) (Figure 7a), an inhibitor of spermidine synthase (Seckute et al., 2011). Together these data suggest that TG muscle overexpressing myostatin showed enhanced spermidine biosynthesis in association with greater mitochondrial respiratory function (Figure 7b).

### 3.7 Myostatin overexpression alters muscle fatty acid composition

Significantly decreased metabolites in enrichment analysis (i.e., for TG–RG/WT–RG) revealed ‘alpha linolenic acid and linoleic acid metabolism’ as the top enriched pathway (Figure 8a). This prompted further examination of individual omega‐6 and omega‐3 polyunsaturated fatty acids (PUFAs) (Figure 8b), bioactive lipid species well‐known to exert control over important biological processes such as inflammation. A majority of omega fatty acids were differentially regulated in TG muscle (Genotype main effects, *P *< 0.05), where both omega‐6 and omega‐3 PUFAs were lower in TG muscle (Figure 8b). TG muscle also had lower saturated fatty acids than WT (Figure 8c), of note because excessive long chain saturated fatty acids like palmitate and stearate are mitotoxic.

To examine how TG and WT responded to metabolic overload by HF diet, metabolites were analysed within each genotype relative to HF diet (i.e., WT–HF/WT–RG and TG–HF/TG–RG). In both genotypes on HF diet, essential fatty acid metabolism was significantly enriched when the increased and decreased metabolites were separately input for enrichment analysis (Appendix Figure A2a,b). From closer inspection of individual metabolites, WT and TG on HF diet both increased omega‐6 but decreased omega‐3 (Figure 8b), consistent with HF diet resulting in increased omega‐6/‐3 ratio to favour a pro‐inflammatory state in muscle. To distinguish features of TG from WT when given the same lipid overload, TG muscle was compared to WT on HF diet (i.e., TG–HF/WT–HF). Here, glycolysis and carbohydrate metabolism were enriched terms when using the metabolites increased in TG muscle on HF diet as input (Appendix Figure A2c), suggesting higher rate of glycolysis (similar to TG muscle on standard diet, Figure 6b). Overall, glycolysis appears to be more active in TG muscle even under different dietary conditions.

When the metabolites that decreased in TG muscle on HF diet were used as input for enrichment (i.e., TG–HF/WT–HF), ‘alpha linolenic acid and linoleic acid metabolism’ was returned as the top pathway (Figure 8d). Further examination showed the omega‐3 fatty acids to be most decreased in TG muscle on HF diet (i.e., TG–HF/WT–HF) (Figure 8b). This suggests the omega‐3 compartment to deplete more severely in TG when challenged by lipid overload, and this feature may distinguish TG muscle (Figure 8e). Depletion of the anti‐inflammatory omega‐3 PUFA compartment could unbalance the ratio of omega‐6/‐3 toward a pro‐inflammatory, functionally limited state (Figure 8e). Given reports that omega‐3 promotes muscle anabolism and OXPHOS (McGlory et al., 2019), deficiency might lower OXPHOS and contribute to more force loss in TG muscle challenged by lipid overload (Figure 5b–h).

In summary, effects of myostatin overexpression under both diet conditions included large differences in glucose metabolism in muscle (Figure 6), as well as spermidine biosynthesis (Figure 7) and lipid composition (Figure 8). Essential and saturated fatty acids decreased in TG muscle (Figure 8) and changed in the opposite direction in null mice (Baati et al., 2017). Myostatin, therefore, has a metabolic regulatory role (e.g., partitioning, oxidation) of carbohydrates, bioactive lipids, polyamines and mitochondrial oxidative function in muscle.

## 4 DISCUSSION

In loss of function experiments using *Mstn* KO or post‐natal inhibition, muscle mass increased but oxidative metabolism was diminished (Mouisel et al., 2014), suggesting that in addition to controlling muscle size, myostatin may also confer high oxidative capacity in muscle. This investigation aimed to examine whether myostatin could promote oxidative capacity in skeletal muscle using a gain of function strategy comprising TG mice that selectively overexpressed myostatin in myofibres. We hypothesized myostatin overexpression in skeletal muscle would promote oxidative metabolism and endurance, and confer tolerance to diet‐induced lipid overload. Findings from our experiments are partially consistent with these hypotheses. TG mice showed strong SDH reactivity in line with oxidative fibre predominance (Figure 2i and Appendix Figure A1d), enhanced in situ OXPHOS capacity and lower fatigability in isolated muscle preparations (Figure 5) and a metabolic profile primed for oxidative metabolism of carbohydrates to sustain muscle performance (Figure 6). Interestingly, these beneficial features at the myofibre level did not clearly translate into better running endurance over WT when using CP and *W*′ as endpoints (Figure 4), presumably because running performance is dependent on multiple physiological mechanisms not exclusively related to skeletal muscle including cardiovascular function and oxygen delivery. Novel regulation of muscle molecular metabolism was also uncovered, where biosynthesis of spermidine, a polyamine compound recently implicated in mitochondrial quality control and geroprotection (Hofer et al., 2022; Madeo et al., 2018), was enhanced by myostatin overexpression (Figure 7). When challenged by 4 weeks of lipid overload, TG muscle responded with reduced insulin sensitivity (Figure 3), greater fatigability, a reduction in OXPHOS (Figure 5b–h) and severely depleted omega‐3 PUFAs (Figure 8) that could favour a pro‐inflammatory environment susceptible to functional limitation. These seemingly detrimental muscle alterations are inconsistent with our hypothesis that higher oxidative capacity in TG muscle would promote tolerance to lipid oversupply; however, these changes did not appear to substantially impact whole animal endurance as running CP was maintained in TG mice on HF diet (Figure 4d). Together, these data position myostatin as a central regulator not just of muscle mass but of metabolism and performance.

A main observation of this study was reduced muscle fatigability and enhanced OXPHOS capacity from muscle‐specific myostatin overexpression (Figure 5), coincident with greater slow fibre expression by SDH staining (Figure 2i and Appendix Figure A1d) and metabolite enrichments reflecting higher oxidative metabolism of carbohydrates (Figure 6). Differentially regulated genes identified on the gene expression panel in KO and TG (Figure 2h), while exploratory and not transcriptome‐wide nor directly proportional to the protein level, might offer preliminary mechanistic insight on how myostatin might regulate muscle metabolism. First, the Fos gene encoding the transcription factor protein c‐Fos was increased in TG but decreased in KO (Figure 2j), highlighting c‐Fos as a potential mechanism of metabolic difference due to myostatin expression status. In cells of the musculoskeletal system, c‐Fos deficiency led to an anaerobic glycolytic phenotype featuring low pyruvate dehydrogenase and high lactate dehydrogenase activities (Matsuoka et al., 2023), whereas restoring pyruvate dehydrogenase activity reversed the anaerobic glycolytic markers seen in c‐Fos deficient cells (Matsuoka et al., 2023). These findings implicate c‐Fos in promoting oxidative capacity and may contribute to the oxidative characteristics seen in TG muscle (Figures 2h,i, 5 and 6). In addition, KO had altered *Pparg* expression while being unchanged in TG (Figure 2j). The peroxisome proliferator‐activated receptor γ coactivator 1α (PPAR) pathway is well known to impact systemic metabolism (Ahmadian et al., 2013; Crossland et al., 2021; Wang et al., 2016). Selective activation of PPARγ in muscle led to an increased proportion of oxidative fibre types (Amin et al., 2010), and suppression of muscle PPARγ  associated with cancer‐induced muscle fatigue and mitochondrial function impairment (Wilson et al., 2019, 2020, 2021), suggesting metabolism in KO versus TG may relate to differential PPARγ activity. In previous work, myostatin was proposed to promote mitochondrial oxidative function by signalling through AMP‐activated protein kinase (AMPK)/SIRT1/peroxisome proliferator‐activated receptor γ coactivator 1 and SMAD2/3 to induce mitochondrial gene expression (Gu et al., 2022; Wang et al., 2022). These pathways may also be active in TG muscle, but we are unable to verify this. Other links to metabolism and mitochondrial function come from the WNT pathway, where both *Wnt1* and *Wnt2* were changed in TG muscle (Figure 2j). Evidence suggested bidirectional cross‐talk between mitochondria and *Wnt*, where WNT signalling stimulated mitochondrial pathways while retrograde communication enabled mitochondrial energy status to alter WNT signalling (Costa et al., 2019; Yoon et al., 2010). Overall, TG muscle showed transcriptional modulation of several signal transduction pathways that could directly affect carbohydrate‐supported mitochondrial ATP provision and muscle performance. Further scrutiny in a larger sample with sensitive high‐throughput sequencing platforms would be a worthwhile step to better define mechanisms in TG muscle.

An unexpected but novel finding was the enrichment of spermidine in TG muscle (Figure 7). This occurred on a regular diet and not through dietary enrichment with spermidine containing foods, indicating the increased spermidine content in TG muscle to arise from myostatin‐associated biosynthesis. The increase in precursor molecules like ornithine coupled with less SAH (Figure 7), an inhibitor of spermidine synthase (Seckute et al., 2011), supports this interpretation and points to upstream signals initiated by myostatin (Durante et al., 2001). Spermidine is a polyamine compound that decreases intracellularly with age (Eisenberg et al., 2009), and extends longevity when supplemented to lower model organisms and human cells (Eisenberg et al., 2009). When spermidine was administered to ageing mice and yeast, oxidative stress and cell death was reduced (Eisenberg et al., 2009). Spermidine pathway molecules also conferred protection against age‐related gut dysfunction and neurodegenerative diseases (Minetti et al., 2025; Sun et al., 2025). In contrast, depletion of endogenous polyamines like spermidine generated oxidative stress, cell death and reduced lifespan (Eisenberg et al., 2009; Mandal et al., 2015), further highlighting the anti‐ageing properties of spermidine. Other potential geroprotective properties of spermidine include pro‐inflammatory cytokine inhibition, autophagy induction, altered fuel metabolism and mitochondrial quality control in association with improvement of ageing‐related phenotypes like hypertension, cognitive impairment, musculoskeletal limitation, heart failure and loss of anti‐cancer immunity (Hofer et al., 2022; Madeo et al., 2018). For instance, aged CD8^+^ T cells that have diminished cytotoxic effector function also featured low spermidine, β‐oxidation, respiration and ATP production (Al‐Habsi et al., 2022). Spermidine treatment reversed these alterations and restored CD8^+^ T cell function (Al‐Habsi et al., 2022), which was mediated by binding and activation of the mitochondrial trifunctional protein (Al‐Habsi et al., 2022). In the heart, spermidine treatment reduced the burden of cardiac ageing and aortic valve disease through modulation of mitochondrial function (Song et al., 2025; Wang et al., 2020). In skeletal muscle, spermidine appears to induce AMPK and CREB to modulate downstream signalling leading to mitochondrial biogenesis and autophagy (Fan et al., 2017; Wang et al., 2021). Based on these collective findings, spermidine revitalizes mitochondrial function and cellular health and informs a plausible mechanism in which myostatin supports muscle homeostasis by linking spermidine synthesis with higher mitochondrial respiration and less muscle fatigability (Figures 5 and 7). This myostatin‐associated shift towards a muscle oxidative phenotype may also have other organismal protective effects that enhance resilience and survival. Evidence from invertebrate models is supportive of this protective role, where *Drosophila* myostatin (myoglianin) prolonged lifespan and delayed systemic ageing through modulation of muscle and adipose (Demontis et al., 2014).

Another prominent finding was the reduction of multiple lipid species in TG muscle, namely the PUFA compartment comprising omega‐6 and omega‐3 fatty acids (Figure 8b), along with saturated fatty acids at long chain lengths (Figure 8c). The lower saturated fat in TG muscle (Figure 8c) is notable because high exposure causes toxicity in musculoskeletal tissues and cell and tissue types of the gastrointestinal and central nervous systems (Brun et al., 2024; Guerbette et al., 2024; Langley et al., 2020; Lin et al., 2024; Pillon et al., 2012; Pinel et al., 2016). This led to mitochondrial fragmentation, respiration impairment, excessive ROS and oxidative stress, endoplasmic reticulum stress, insulin resistance and metabolic disease risk (Guerbette et al., 2024; Putti et al., 2015). Lower saturated fat, therefore, may have favoured the higher respiration rates seen in TG muscle (Figure 5). Indeed, reduced exposure to the saturated fatty acid palmitate enhanced mitochondrial metabolism in hepatocytes (Liu et al., 2020), a high energy demand tissue similar to muscle. Unlike saturated fats, PUFAs are essential nutrients that must be obtained from dietary intake. As bioactive lipids, PUFAs regulate cell functions such as signal transduction and gene expression, inflammation and immunity and are implicated in multiple forms of illness such as cancer, cardiovascular and neurodegenerative diseases (Lee‐Okada et al., 2025). Because omega‐6 and omega‐3 PUFAs are generally pro‐inflammatory and anti‐inflammatory, respectively, their relative intake is considered an indicator of nutritional adequacy and disease risk (Cao et al., 2024; Mariamenatu & Abdu, 2021; Scaioli et al., 2017; Simopoulos, 2002). Disproportionate consumption of omega‐6 over omega‐3 tips the balance toward a pro‐inflammatory state that when persistent is believed to contribute to chronic disease susceptibility (Figure 8e) (Cao et al., 2024; Mariamenatu & Abdu, 2021; Scaioli et al., 2017; Simopoulos, 2002). An increase in omega‐6/‐3 is typical of western diets rich in saturated fat and low on fruits and vegetables (Blasbalg et al., 2011; Mariamenatu & Abdu, 2021; Simopoulos, 2002), consistent with both WT and TG mice showing higher omega‐6, lower omega‐3 and elevated saturated fatty acids after HF diet (Figure 8b,c,e). Interestingly, TG muscle showed a strong interaction with diet, where HF diet exacerbated omega‐3 fatty acid depletion (Figure 8b). Depleted omega‐3 might partially explain some of the poor responses on HF diet of TG mice, which showed reduced insulin sensitivity (Figure 3), muscle fatigability and OXPHOS limitation (Figure 5b–h). In prior work, omega‐3 fatty acid intake had overall positive effects on muscle as reflected by improvement in insulin sensitivity and signalling, mitochondrial OXPHOS, oxidative stress and protein turnover (Gui et al., 2020; Lepretti et al., 2018; McGlory et al., 2019; Pinel et al., 2016; Smith, 2016). Further, muscle is selectively enriched in omega‐3 and particularly sensitive to dietary fatty acid based interventions (Zhang et al., 2023). It is therefore conceivable that myostatin overexpression conferred hypersensitivity to HF diet, leading to excessive omega‐3 depletion in muscle and some of the detrimental outcomes in TG mice. Despite some of these apparent decrements at the muscle level, only TG mice maintained CP after HF diet (Figure 4d), suggesting that myostatin maintained or supported other physiological systems relevant to whole animal exercise endurance.

The mechanism by which myostatin altered PUFA and saturated fat in TG muscle (Figure 8) could not be extensively studied in the present dataset due to limited protein level measurements. Because TG mice consumed a standard, controlled diet not different in quantity from WT (Figure 1d), and since TG physical activity levels were also not significantly different (Figure 1e), the lowered fatty acids in TG may have occurred mainly from altered endogenous metabolism due to myostatin overexpression. Although enhanced lipid oxidation is a logical explanation, the metabolite profile of TG muscle was inconclusive and did not unambiguously indicate higher rates of lipid oxidation (Supporting information Table S1). Transcript expression of the gene encoding fatty acid synthase (*Fasn*) was decreased in TG and this might contribute to fewer long chain saturated fatty acids (Figure 2j). In previous work on the lipidome in *Mstn* KO mice, an opposing phenotype was reported where KO muscle had greater saturated fat, and more total PUFA driven by increases in both omega‐6 and omega‐3 classes (Baati et al., 2017). Natural candidates for these observed effects are enzymes of the fatty acid synthesis pathway. Delta‐5 and delta‐6 desaturases are key rate‐limiting enzymes in PUFA metabolism. Delta‐5 desaturase, encoded by the *Fads1* gene, is responsible for generating arachidonic acid in the omega‐6 pathway and eicosapentaenoic acid in the omega‐3 pathway. Delta‐6 desaturase, encoded by *Fads2*, synthesizes the omega‐3s eicosapentaenoic acid and docosahexaenoic acid from α‐linolenic acid. Although concentration of delta‐5 desaturase was not different in KO mice versus WT (Baati et al., 2017), other desaturase enzymes were reported altered in KO animals (Baati et al., 2017; Ren et al., 2020), where one proposed mechanism involved the muscle‐specific transcription factor MEF2C (Ren et al., 2020). Based on these studies, a plausible interpretation of our findings is that in TG muscle, myostatin initiated a signalling cascade that altered the expression and/or activity of desaturases and other enzymes of the fatty acid synthesis pathway to maintain a lower muscle lipid profile.

There are several limitations of the present work. A direct examination of muscle fibre type to detect myosin heavy chain (MHC) isoforms on transverse sections was not performed. While the SDH stain provides a marker of oxidative phenotype, and strong SDH reactivity was reported to correlate best with slow MHC fibre types (Blemker et al., 2024; Rivero et al., 1999), it does not identify specific shifts in fibre type composition (i.e., I, IIA, IIX, IIB and hybrids). Thus, we are unable to address whether myostatin overexpression may promote its oxidative fibre phenotype in TG mice through altered fibre type expression, particularly in the promotion of slow type I and IIA fibres that have greater oxidative activity. Another limitation is that the mechanisms we have proposed to underlie greater in situ respiration and fatigue resistance in TG mice, such as polyamine‐related mitochondrial quality control, are derived exclusively from the metabolome analysis. In this instance, the readout only allows for an association of underlying mechanisms to be speculated, but not the establishment of cause and effect. Further experiments that directly manipulate and assess regulators of polyamines and mitochondrial quality control are needed to investigate the mechanistic potential of spermidine and mitophagy pathways in myostatin‐dependent oxidative metabolism in muscle. We chose to investigate the hypothesized resistance to HF diet using a mitochondrial respiratory protocol that probed fatty acid oxidation using palmitoyl‐carnitine as a substrate. Given our metabolomic findings suggesting enhanced carbohydrate‐fuelled OXPHOS as a consequence of muscle‐specific myostatin overexpression, a follow‐up respirometry experiment with pyruvate‐based respiratory substrates would have been useful to investigate if TG muscle had greater carbohydrate‐fuelled OXPHOS. In addition, our interpretation of muscle fatigability was that TG and KO demonstrated sensitivity to the HF diet, as reflected by greater force loss at stimulation frequencies of 30, 60 and 120 Hz. This loss of force, however, was not as pronounced at 15 Hz. This may suggest that muscle fatigability in TG and KO were not sensitive to HF at lower stimulation frequencies that result in lower absolute forces.

In summary, these data offer evidence that transgenic, muscle‐selective myostatin overexpression promoted oxidative phosphorylation capacity and fatigue resistance in skeletal muscle. These indicators of improved mitochondrial and contractile function were coupled to a metabolite profile reflecting bioenergetic priming for carbohydrate‐fuelled oxidative metabolism to support oxidative muscle ATP provision. Marked remodelling of muscle lipid composition occurred in which there was a lowering of saturated fatty acids and PUFA in myostatin overexpressing muscle. Novel regulation of muscle molecular metabolism was also uncovered through enhanced synthesis of spermidine, a polyamine compound with demonstrated regulation of β‐oxidation, mitochondrial respiratory function, protein quality control and overall geroprotection, thus informing a potential mechanism in which myostatin supports muscle homeostasis by linking spermidine synthesis with higher mitochondrial respiration and less muscle fatigability. When challenged by lipid overload, myostatin overexpressing muscle showed reduced insulin sensitivity, more force loss and lower OXPHOS and excessive depletion of omega‐3 fatty acids but also signs of resilience based on indicators of mitochondrial respiratory control and running CP. Together, these data are consistent with myostatin acting not only as a significantly regulator of muscle mass but also as a central mediator controlling diverse metabolic pathways in muscle.

## AUTHOR CONTRIBUTIONS

Conception or design of the work: Andy V. Khamoui, Andrea Abraham, Janos Porszasz, Istvan Kovanecz, Silvana Constantinescu, Harry B. Rossiter and Suzanne Reisz‐Porszasz. Acquisition, analysis or interpretation of data for the work: Andy V. Khamoui, Andrea Abraham, Janos Porszasz, Istvan Kovanecz, Silvana Constantinescu, Harry B. Rossiter and Suzanne Reisz‐Porszasz. Drafting the work or revising it critically for important intellectual content: Andy V. Khamoui, Andrea Abraham, Janos Porszasz, Istvan Kovanecz, Silvana Constantinescu, Harry B. Rossiter and Suzanne Reisz‐Porszasz. Experiments were conducted at Charles R. Drew University of Medicine and Science, with exception for respiration experiments which were performed at The Lundquist Institute for Biomedical Innovation at Harbor‐UCLA Medical Center, and blood biochemistry performed at the Mouse Metabolic Phenotyping Center at the University of Cincinnati.

Andy V. Khamoui, Andrea Abraham, Janos Porszasz, Istvan Kovanecz, Silvana Constantinescu, Harry B. Rossiter and Suzanne Reisz‐Porszasz approved the final version of the manuscript, agree to be accountable for all aspects of the work in ensuring that questions related to the accuracy or integrity of any part of the work are appropriately investigated and resolved; and confirm that all persons designated as authors qualify for authorship and all those who qualify for authorship are listed.

## CONFLICT OF INTEREST

The authors declare that there is no conflict of interest that would prejudice the impartiality of this scientific work. H.B.R. reports consulting fees from the NIH RECOVER‐ENERGIZE working group (1OT2HL156812) and is involved in contracted clinical research with Biocient, Intervene Immune, Mezzion, Regeneron, Respira and Roche.

## Supporting information



Table S1 Muscle Metabolite Analysis

## Data Availability

All data supporting the results are presented in the manuscript.
